# Cost‐effectiveness of statins for primary prevention of atherosclerotic cardiovascular disease among people living with HIV in the United States

**DOI:** 10.1002/jia2.25690

**Published:** 2021-03-21

**Authors:** David C Boettiger, Anthony T Newall, Andrew Phillips, Eran Bendavid, Matthew G Law, Lene Ryom, Peter Reiss, Amanda Mocroft, Fabrice Bonnet, Rainer Weber, Wafaa El‐Sadr, Antonella d’Arminio Monforte, Stephane de Wit, Christian Pradier, Camilla I Hatleberg, Jens Lundgren, Caroline Sabin, James G Kahn, Dhruv S Kazi

**Affiliations:** ^1^ Philip R. Lee Institute for Health Policy Studies University of California San Francisco CA USA; ^2^ Kirby Institute UNSW Sydney Sydney NSW Australia; ^3^ The School of Public Health and Community Medicine UNSW Sydney Sydney NSW Australia; ^4^ Institute for Global Health University College London London UK; ^5^ Center for Health Policy and the Center for Primary Care and Outcomes Research Stanford University Stanford CA USA; ^6^ Rigshospitalet University of Copenhagen Copenhagen Denmark; ^7^ Amsterdam University Medical Centers University of Amsterdam Amsterdam The Netherlands; ^8^ HIV Monitoring Foundation Amsterdam The Netherlands; ^9^ Université Bordeaux CHU de Bordeaux France; ^10^ University Hospital Zurich University of Zurich Zurich Switzerland; ^11^ ICAP‐Columbia University and Harlem Hospital New York NY USA; ^12^ Clinica di Malattie Infettive e Tropicali Azienda Ospedaliera‐Polo Universitario San Paolo Milan Italy; ^13^ Saint Pierre University Hospital Université Libre de Bruxelles Brussels Belgium; ^14^ Department of Public Health Nice University Hospital Nice France; ^15^ Smith Center for Outcomes Research in Cardiology Beth Israel Deaconess Medical Center Boston MA USA; ^16^ Harvard Medical School Harvard University Boston MA USA

**Keywords:** HIV, cardiovascular disease, statin, cost‐effectiveness, United States, antiretroviral therapy

## Abstract

**Background:**

Expanding statin use may help to alleviate the excess burden of atherosclerotic cardiovascular disease in people living with HIV (PLHIV). Pravastatin and pitavastatin are preferred agents due to their lack of substantial interaction with antiretroviral therapy. We aimed to evaluate the cost‐effectiveness of pravastatin and pitavastatin for the primary prevention of atherosclerotic cardiovascular disease among PLHIV in the United States.

**Methods:**

We developed a microsimulation model that randomly selected (with replacement) individuals from the Data‐collection on Adverse Effects of Anti‐HIV Drugs study with follow‐up between 2013 and 2016. Our study population was PLHIV aged 40 to 75 years, stable on antiretroviral therapy, and not currently using lipid‐lowering therapy. Direct medical costs and quality‐adjusted life‐years (QALYs) were assigned in annual cycles and discounted at 3% per year. We assumed a willingness‐to‐pay threshold of $100,000/QALY gained. The interventions assessed were as follows: (1) treating no one with statins; (2) treating everyone with generic pravastatin 40 mg/day (drug cost $236/year) and (3) treating everyone with branded pitavastatin 4 mg/day (drug cost $2,828/year). The model simulated each individual’s probability of experiencing atherosclerotic cardiovascular disease over 20 years.

**Results:**

Persons receiving pravastatin accrued 0.024 additional QALYs compared with those not receiving a statin, at an incremental cost of $1338, giving an incremental cost‐effectiveness ratio of $56,000/QALY gained. Individuals receiving pitavastatin accumulated 0.013 additional QALYs compared with those using pravastatin, at an additional cost of $18,251, giving an incremental cost‐effectiveness ratio of $1,444,000/QALY gained. These findings were most sensitive to the pill burden associated with daily statin administration, statin costs, statin efficacy and baseline atherosclerotic cardiovascular disease risk. In probabilistic sensitivity analysis, no statin was optimal in 5.2% of simulations, pravastatin was optimal in 94.8% of simulations and pitavastatin was never optimal.

**Conclusions:**

Pravastatin was projected to be cost‐effective compared with no statin. With substantial price reduction, pitavastatin may be cost‐effective compared with pravastatin. These findings bode well for the expanded use of statins among PLHIV in the United States. To gain greater confidence in our conclusions it is important to generate strong, HIV‐specific estimates on the efficacy of statins and the quality‐of‐life burden associated with taking an additional daily pill.

## 1 INTRODUCTION

People living with HIV (PLHIV) have an elevated risk of atherosclerotic cardiovascular disease (ASCVD) compared to people without HIV [1]. This is only partially explained by the high prevalence of cardiovascular risk factors among PLHIV. In a landmark study of 82,459 participants, those who were HIV‐infected had a 48% increased risk of incident type 1 myocardial infarction (T1MI) compared with uninfected participants, even after adjusting for traditional risk factors, comorbidities and substance use [2]. Similar studies have also found a significant increase in other ASCVD outcomes associated with HIV infection [3, 4]. The increased ASCVD risk associated with HIV may be mediated by the virus itself, past or present immunodeficiency, adverse effects of antiretroviral therapy (ART) or a combination of these factors [5]. There is also evidence that PLHIV are less likely than uninfected persons to receive recommended statin therapy [6].

Statins reduce ASCVD risk primarily by lowering low‐density lipoprotein (LDL) cholesterol [7]. They also have anti‐inflammatory properties that may enhance their effectiveness in PLHIV [8]. The current American College of Cardiology/American Heart Association guidelines state that HIV is a risk‐enhancing factor that may favour statin initiation among people at intermediate risk of ASCVD [9].

Several studies have suggested that wider use of statins in the general US population could be cost‐effective [10, 11]. However, most members of the general population are unaccustomed to taking regular medication. PLHIV on ART appear to exhibit better statin adherence than the general population [12]. Hence, a policy of universally recommending statins to PLHIV of a certain age may achieve greater acceptance. It could also simplify advice and substantially reduce ASCVD rates.

Nevertheless, expanding the use of statins in the context of high rates of ART coverage is challenging because of drug interactions leading to intolerance or reduced efficacy. With concomitant protease inhibitor use, simvastatin and lovastatin are contraindicated, whereas atorvastatin and rosuvastatin require modified dosing [13]. With concomitant efavirenz or etravirine use, statins may require dose modification [13]. Pravastatin and pitavastatin are preferred agents because they improve cholesterol levels and reduce immune activation without interacting substantially with ART [14, 15, 16]. While pitavastatin is still under patent and substantially more expensive than other statins, current evidence suggests it produces greater improvements in cholesterol levels than pravastatin among PLHIV [14].

Given these trade‐offs, we evaluated the cost‐effectiveness of pravastatin and pitavastatin for the primary prevention of ASCVD among PLHIV in the United States. This work is a companion piece to our earlier work addressing the same research question in the Thai HIV population [17].

## 2 METHODS

### 2.1 Study population, model structure, model parameterization and model validation

We developed a model that randomly selected (with replacement) individuals from the Data‐collection on Adverse Effects of Anti‐HIV Drugs (D:A:D) study with follow‐up between 2013 and 2016 who, at their last clinic visit, were aged 40 to 75 years, had no history of ASCVD, were not using lipid‐lowering therapy, had been using ART for at least six months, and had a CD4 cell count >100 cells/mm^3^. Primary ASCVD risk was calculated using the reduced D:A:D CVD risk equation [18]. Non‐CVD mortality rates were based on those of the general population as PLHIV on stable ART have been shown to have comparable rates [19]. The model assumed the US healthcare sector perspective and applied a 20‐year time horizon to allow sufficient event accumulation to compare treatment strategies. All cohorts in D:A:D follow local national guidelines and regulations regarding participant consent and ethical review. This analysis was approved by both the University of California, San Francisco Institutional Review Board (IRB#18‐25654) and the UNSW Sydney Human Research Ethics Committee Executive (HC#180398). Further details on the study population, model structure and model parameterization are available in the Tables S1 to S12 and Figure S1).

**FIGURE 1:**
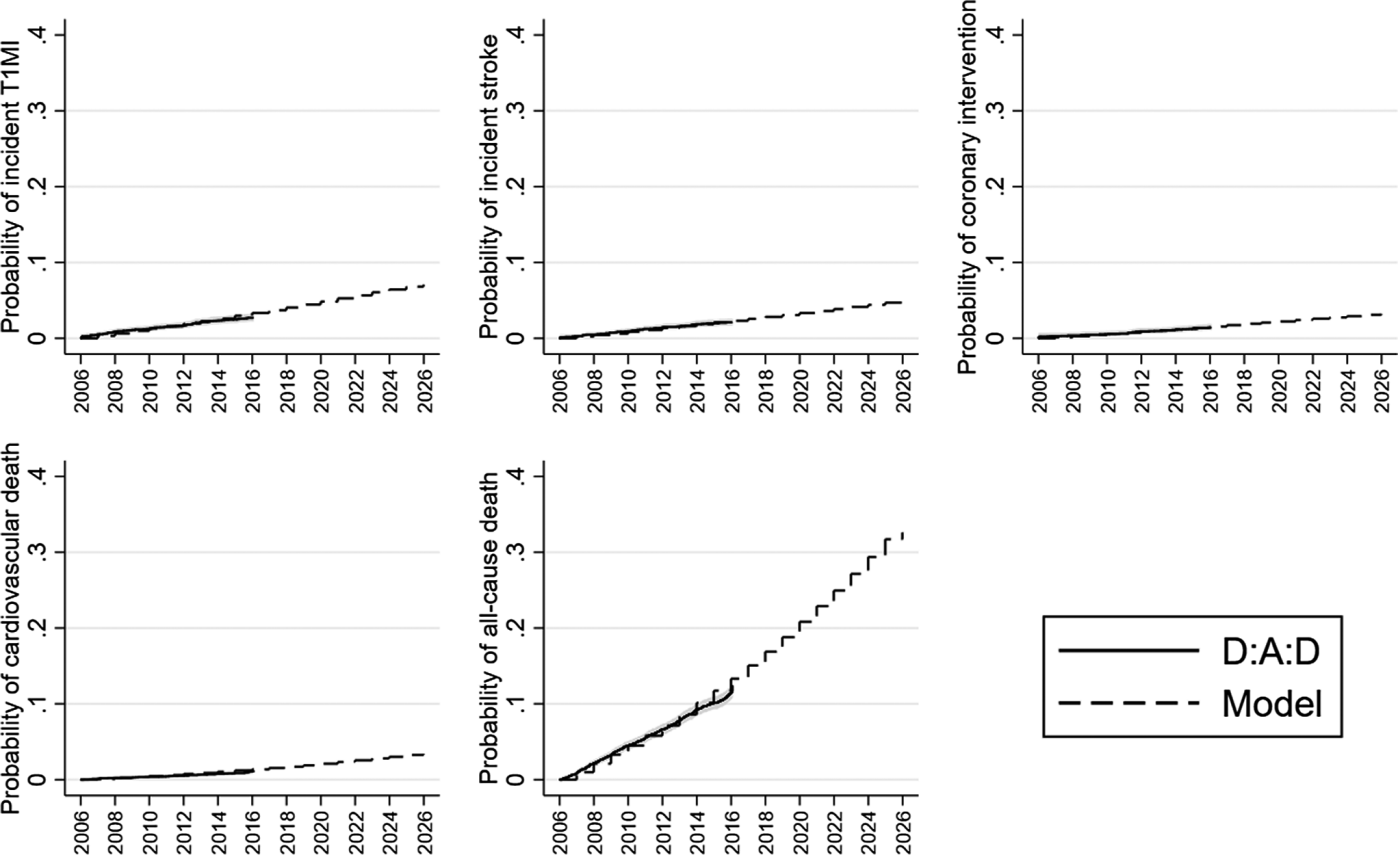
Observed versus modelled probability of incident T1MI, incident stroke, coronary intervention, cardiovascular death and all‐cause death over time. Observed data is from the Data‐collection on Adverse Effects of Anti‐HIV Drugs (D:A:D) study. Shaded area is 95% confidence interval for observed data. T1MI, type 1 myocardial infarction.

We calibrated our model based on the observed rates of coronary intervention, incident T1MI, incident stroke, cardiovascular death and all‐cause death seen among D:A:D participants between 2006 and 2016. Figure 1 shows that our calibrated model estimates provided an accurate reflection of the observed data.

### 2.2 Treatment strategies

We evaluated three treatment strategies: (1) treating none of the study population with a statin; (2) treating the entire study population with pravastatin 40 mg/day and (3) treating the entire study population with pitavastatin 4 mg/day. During the first year of statin use, we assumed individuals would achieve the same reductions in LDL cholesterol observed in a recent clinical trial among PLHIV (20.5% for pravastatin; 29.7% for pitavastatin) [14]. Thereafter, statin adherence, and hence LDL lowering efficacy and associated adverse event rates, were reduced by 50% [20, 21, 22]. The reduction in ASCVD risk associated with each individual’s mmol/L LDL reduction was varied by five‐year CVD risk (based on the D:A:D equation) and ASCVD event (Table S11) [7]. We assumed PLHIV would only incur the cost of statins they were using and hence statin costs were reduced by 50% after the first year, in line with the assumed decline in adherence.

In sensitivity analyses, we assumed statins produce additional ASCVD preventative efficacy among PLHIV mediated by their anti‐inflammatory properties [8]. We assumed that statins do not prevent non‐ASCVD events as current literature suggests little or no benefit for such outcomes [23]. We modelled adverse events related to statin use (haemorrhagic stroke, diabetes and myopathy) based on rates observed in the general population. Unfortunately, studies of sufficient size to accurately determine the incidence of serious statin adverse events in PLHIV have not been conducted. Although atorvastatin and rosuvastatin are often used with caution in PLHIV [24], we did not explicitly model these statins as their efficacies and costs are comparable to, and within the sensitivity ranges adopted for, pravastatin [15, 25]. It is reasonable to view our pravastatin arm as a generic atorvastatin and/or generic rosuvastatin arm under the assumption that the ART interaction profiles of atorvastatin and rosuvastatin are acceptable to PLHIV who are mostly at low to moderate risk of ASCVD. It may also be acceptable to use fluvastatin with caution in PLHIV, although it rarely is in practice [24], and would be less cost‐effective than pravastatin due to its similar efficacy and greater cost [15, 25]. Hence, we did not include a fluvastatin arm in our analysis.

### 2.3 Cost and quality‐of‐life estimates

Health‐related costs and quality‐of‐life (or health state utility) adjustments were assigned to each clinical event and health state in annual cycles. We included all direct medical costs regardless of who paid for them. Cost estimates were inflated to 2019 US dollars using the US Bureau of Labor Statistics Consumer Price Index Inflation Calculator [26]. Annual drug costs were assumed to be equivalent to the wholesale acquisition cost of branded pitavastatin 4 mg/day ($2,828) and the median cost of generic formulations of pravastatin 40 mg/day ($236) [25]. We varied the cost of pitavastatin widely in sensitivity analyses because a price reduction is likely when generic products become available (expected between 2021 and 2025). Since people using ART are already required to take at least one daily pill, we assumed that remembering to take a daily statin and the inconvenience of doing so (pill burden) was not associated with any quality‐of‐life decrement. Utilities were calculated using a multiplicative approach in those with multiple comorbidities, except for individuals with a history of both ischaemic and haemorrhagic stroke for whom we assumed a utility burden consistent with haemorrhagic stroke (i.e. a minimum approach). Future costs and benefits were discounted at 3% per year [27].

### 2.4 Outcomes

The primary outcome was the incremental cost‐effectiveness ratio (ICER; defined as the cost per QALY gained). The threshold for an intervention being deemed cost‐effective was defined as an ICER below $100,000/QALY gained (willingness‐to‐pay threshold) [28]. Our secondary outcomes included incremental QALYs gained, incremental costs incurred, incremental life‐years gained and the incremental cost per life‐year gained.

### 2.5 Sensitivity analyses

We used sensitivity analyses to evaluate the robustness of our results to uncertainty in key input parameters. In deterministic sensitivity analyses, we varied one or two input parameters at a time while holding others constant at their base‐case estimates. In probabilistic sensitivity analyses, we varied multiple input parameters across prespecified distributions over 500 iterations. Beta distributions were used for utilities and event probabilities, and log‐normal distributions were used for hazard ratios, safety and efficacy measures and costs.

### 2.6 Scenario analyses

In addition to our sensitivity analyses, we investigated the following scenarios:

1. Restricting the study population to PLHIV at >1% risk of CVD in the next five years (as defined by the D:A:D equation) such that those with ≤1% risk were excluded from the analysis. We assumed no additional costs for ASCVD risk factor screening as this is standard practice for PLHIV in the United States;
2. Restricting the study population to PLHIV at >5% risk of CVD in the next five years (as defined by the D:A:D equation) such that those with ≤5% risk were excluded from the analysis. As for the above scenario, we assumed no additional cost for ASCVD risk factor screening;
3. Using the revised 2013 pooled cohort equations [29] instead of the D:A:D equation to calculate T1MI and ischaemic stroke risk and applying a prespecified level of additional risk associated with being HIV positive. The pooled cohort equations are well validated in the general US population. However, risk equations based on the general population have been shown to regularly underestimate ASCVD risk in PLHIV [30, 31]. We assumed PLHIV in our analysis were at a 48% (range for sensitivity testing 27% to 97%) increased risk of T1MI [2], and a 17% (range for sensitivity testing 1% to 36%) increased risk of ischaemic stroke [3] than estimated by the pooled cohort equations.
4. Assuming the probability of ASCVD while using pravastatin and pitavastatin was reduced by a further 10% and 20%, respectively, to account for the possibility that pitavastatin exhibits greater additional efficacy associated with reducing inflammation. Pitavastatin has been shown to produce greater reductions in important inflammatory markers compared with pravastatin [16].

### 2.7 Software

Data management and statistical analysis were conducted using SAS 9.4 (SAS Institute Inc). Modelling was performed in TreeAge Pro 2020 Version R1.0 (TreeAge Software).

## 3 RESULTS

### 3.1 Base‐case analysis

Modelled rates for incident T1MI, incident ischaemic stroke and fatal CVD among the no statin group were 4.4, 2.3 and 1.9 per 1000 person‐years respectively. The all‐cause mortality rate in the no statin group was 18.5 per 1000 person‐years and, over the next 20 years, individuals were projected to accumulate a discounted average of 13.455 QALYs, 13.584 life‐years and $478,454 in direct medical costs (Table 1).

**Table 1:** Incremental cost‐effectiveness of pravastatin and pitavastatin for primary prevention of ASCVD among PLHIV

Intervention	Total cost (inclusive of statin cost), $	Statin cost, $	Incident type 1 T1MI^a^	Incident Ischaemic stroke^a^	Fatal CVD^a^	All‐cause mortality^a^	Life‐years	QALYs	Incremental cost, $	Incremental life‐years gained	Incremental QALYs gained	$/life‐year gained^b^	ICER, $/QALY gained^b^
Base‐case													
None	478,454	0	4.4	2.3	1.9	18.5	13.584	13.455	–	–	–	–	–
Pravastatin 40 mg	479,792	3097	4.0	2.1	1.8	18.3	13.596	13.478	1338	0.012	0.024	111,000	56,000
Pitavastatin 4 mg	498,043	37,207	3.8	2.0	1.7	18.3	13.603	13.491	18,251	0.007	0.013	2,688,000	1,444,000
Scenario 1) Restricting the study population to PLHIV at > 1% risk of CVD in the next five years													
None	477,798	0	4.8	2.5	2.0	19.7	13.485	13.345	–	–	–	–	–
Pravastatin 40 mg	479,172	3066	4.2	2.3	1.9	19.6	13.499	13.372	1374	0.014	0.027	98,000	51,000
Pitavastatin 4 mg	497,363	36,842	3.9	2.3	1.8	19.5	13.509	13.388	18,191	0.010	0.016	1,806,000	1,163,000
Scenario 2) Restricting the study population to PLHIV at > 5% risk of CVD in the next five years													
None	466,463	0	7.9	4.3	3.5	28.8	12.704	12.469	–	–	–	–	–
Pravastatin 40 mg	467,899	2802	7.1	4.1	3.2	28.5	12.731	12.515	1,436	0.027	0.046	53,000	31,000
Pitavastatin 4 mg	484,515	33,724	6.8	3.9	3.1	28.4	12.742	12.536	16,616	0.012	0.021	1,442,000	805,000
Scenario 3) Using the pooled cohort equations instead of the D:A:D equation to calculate T1MI and ischaemic stroke risk													
None	478,905	0	6.2	2.6	2.3	18.8	13.551	13.393	–	–	–	–	–
Pravastatin 40 mg	480,335	3064	5.5	2.4	2.1	18.7	13.569	13.425	1430	0.017	0.032	83,000	45,000
Pitavastatin 4 mg	498,420	36,828	5.3	2.3	2.0	18.6	13.578	13.441	18,085	0.009	0.016	1,945,000	1,148,000
Scenario 4) Assuming pravastatin and pitavastatin exhibit 10% and 20% additional efficacy respectively^c^													
None	478,454	0	4.4	2.3	1.9	18.5	13.584	13.455	–	–	–	–	–
Pravastatin 40 mg	479,645	3105	3.8	2.0	1.7	18.3	13.603	13.491	1191	0.019	0.037	64,000	33,000
Pitavastatin 4 mg	497,907	37,370	3.4	1.8	1.5	18.1	13.617	13.516	18,262	0.015	0.024	1,258,000	748,000

Incremental cost‐effectiveness for each strategy was measured relative to the next best strategy in terms of QALYs gained (i.e. pravastatin vs. no statin, and pitavastatin vs. pravastatin); Costs, QALYs, and life‐years were discounted at 3%/year. ASCVD, atherosclerotic cardiovascular disease; D:A:D, Data‐collection on Adverse Effects of Anti‐HIV Drugs; ICER, incremental cost‐effectiveness ratio; PLHIV, people living with HIV; QALY, quality‐adjusted life‐year; T1MI, type 1 myocardial infarction.

^a^ Per 1000 person‐years

^b^ rounded to nearest thousand

^c^ the base‐case probability of ASCVD while using pravastatin and pitavastatin was reduced by 10% and 20%, respectively, to account for the possibility that pitavastatin exhibits greater additional efficacy associated with reducing inflammation.

Compared with PLHIV in the no statin arm, those receiving pravastatin had a 9.1%, 8.7% and 5.3% reduction in the rate of incident T1MI, ischaemic stroke and fatal CVD, respectively, and accrued 0.024 additional QALYs at an incremental cost of $1,338, resulting in an ICER of $56,000/QALY gained. Compared with PLHIV in the pravastatin arm, those receiving pitavastatin had a 5.0%, 4.8% and 5.6% reduction in the rate of incident T1MI, incident ischaemic stroke and fatal CVD, respectively, and accumulated 0.013 additional QALYs at an incremental cost of $18,251, giving an ICER of $1,444,000/QALY gained (Table 1).

### 3.2 Sensitivity analyses

In our base‐case analysis, we assumed that the pill burden associated with daily statin use did not cause any quality‐of‐life decrement. When a decrement was assumed, the average number of QALYs accumulated in the active treatment arms was reduced substantially. At the upper bound of our sensitivity range (0.00384 QALYs lost per year, the equivalent of losing four weeks of perfect health over 20 years [11]), both pravastatin and pitavastatin resulted in a net QALY loss compared to no statin use. At a decrement of ≥0.00136, pravastatin ceased to be cost‐effective compared to no statin at a willingness‐to‐pay threshold of $100,000/QALY gained.

**FIGURE 2:**
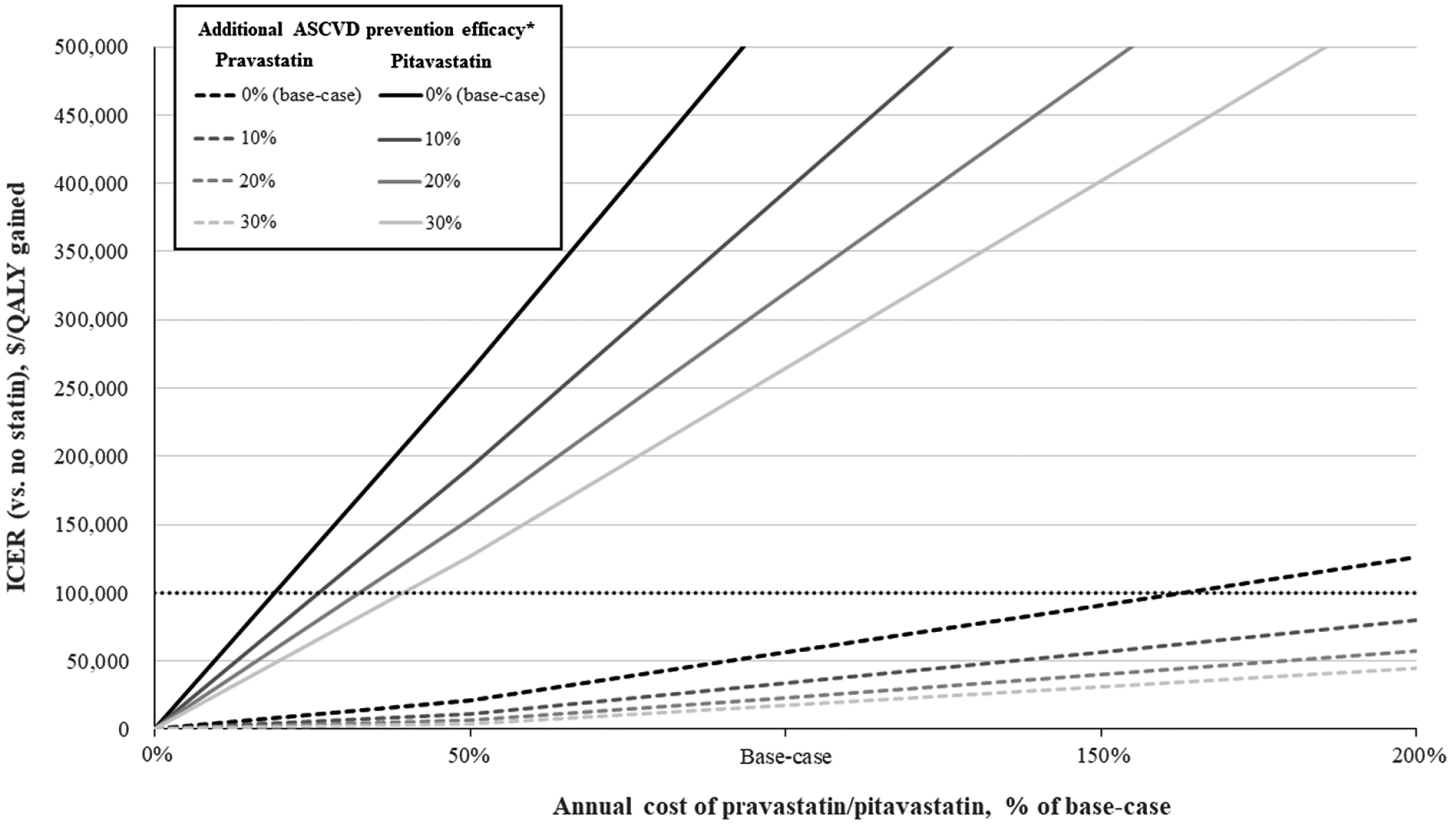
ICERs for pravastatin vs. no statin and pitavastatin vs. no statin under various assumptions for statin cost and additional ASCVD prevention efficacy^a^. Horizontal dashed line represents an ICER of $100,000/QALY gained; ^a^The base‐case probability of ASCVD while using pravastatin was reduced by various percentages to account for the possibility of preventative efficacy associated with the anti‐inflammatory properties of statins in PLHIV; ICER, incremental cost‐effectiveness ratio; ASCVD, atherosclerotic cardiovascular disease; QALY, quality‐adjusted life‐year; PLHIV, people living with HIV.

Our results were also sensitive to changes in drug cost and drug efficacy. If the annual cost of pravastatin was increased to ≥$384 (163% of base‐case price), it ceased to be cost‐effective compared to no statin. When the probability of ASCVD while using pravastatin was reduced by 30% to account for the possibility of additional preventative efficacy associated with the anti‐inflammatory properties of statins, the ICER dropped to $18,000/QALY gained compared to no statin. Figure 2 shows the results of a two‐way sensitivity analysis where our estimates of pravastatin price and additional ASCVD preventative efficacy were varied. In the most pessimistic case, where we assumed the annual price of pravastatin was $472 and that statins do not exhibit ASCVD preventative efficacy associated with their anti‐inflammatory properties, the ICER was $126,000/QALY gained. In the most optimistic case, where we assumed the annual price of pravastatin was $118 and the probability of ASCVD while using pravastatin was reduced by 30%, the ICER was $4,000/QALY gained.

A time horizon shorter than the base‐case of 20 years resulted in less favourable ICERs for both the pravastatin versus no statin and pitavastatin versus pravastatin comparisons. When the time horizon was ≤12 years, pravastatin did not meet our definition for being cost‐effective when compared to no statin. Other model parameters had a smaller effect on our base‐case results when varied in one‐way sensitivity analyses (Figures S2 and S3)

Given the price of pitavastatin is likely to drop more substantially than that of pravastatin in coming years, and that pitavastatin may be preferred over other statins for its favourable interaction profile with ART [13], we also compared pitavastatin to no statin with varying drug prices and additional ASCVD preventative efficacy. Even with 30% additional ASCVD preventative efficacy, the price of pitavastatin needed to drop below 50% of the base‐case price to become cost‐effective compared with no statin (Figure 2). If the annual cost of pitavastatin was reduced to <$350 (12.4% of the base‐case price), the pitavastatin versus no statin ICER became better than our base‐case pravastatin versus no statin ICER (i.e. pitavastatin became dominant (extended) over pravastatin; Figure S3).

**FIGURE 3:**
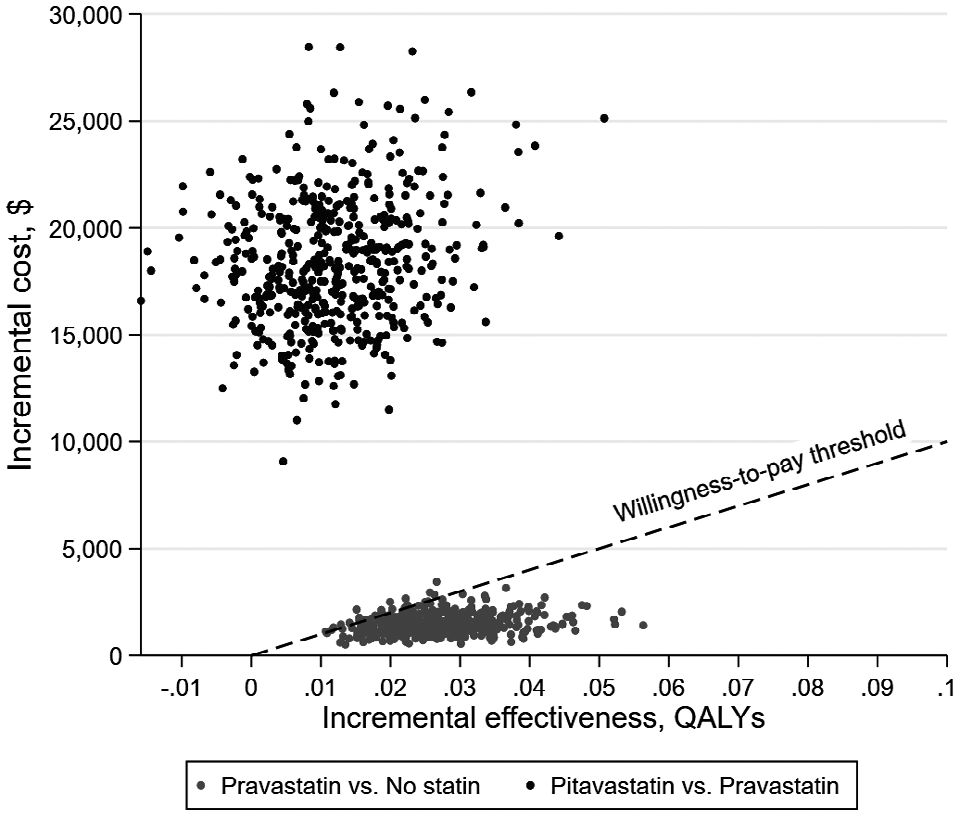
Incremental effectiveness vs. incremental cost over 500 probability sensitivity analysis simulations. Dashed line represents willingness‐to‐pay threshold of $100,000/QALY gained. Dots below the willingness‐to‐pay line indicate the intervention is cost‐effective relative to the comparator, while dots above the line indicate the intervention is not cost‐effective relative to the comparator.

In our probabilistic sensitivity analysis, no statin was optimal in 5.2% of simulations at a willingness‐to‐pay of $100,000/QALY gained, pravastatin was optimal in 94.8% of simulations and pitavastatin was never optimal (Figure 3).

### 3.3 Scenario analyses

The results of our scenario analyses are displayed in Table 1. Treating only PLHIV at >1% risk of CVD in the next five years (scenario 1) improved the ICER for pravastatin versus no statin to $51,000/QALY gained and the ICER for pitavastatin versus pravastatin to $1,163,000/QALY gained. Further restricting statin therapy to only those at >5% risk of CVD in the next five years (scenario 2) improved the ICER for pravastatin versus no statin to $31,000/QALY gained, and the ICER for pitavastatin versus pravastatin to $805,000/QALY gained.

When using the pooled cohort equations in place of the D:A:D equation to calculate T1MI and ischaemic stroke risk (scenario 3), our model predicted higher rates of incident T1MI, incident ischaemic stroke and fatal CVD compared with the base‐case. This led to more favourable ICERs for pravastatin versus no statin (45,000/QALY gained) and for pitavastatin versus pravastatin ($1,148,000/QALY gained). Similar results were observed in sensitivity analyses when the additional risks associated with being HIV positive were varied for T1MI and ischaemic stroke.

## 4 DISCUSSION

At current drug prices, we estimated that expanding pravastatin use to PLHIV aged 40 to 75 years, stable on ART, and not currently using lipid‐lowering therapy would be cost‐effective for the primary prevention of ASCVD at a willingness‐to‐pay threshold of $100,00/QALY gained. Pitavastatin would require substantial price reduction before becoming cost‐effective when compared to pravastatin or no statin. These findings were sensitive to the pill burden associated with daily statin administration, statin costs and statin efficacy, but robust across a wide range of other sensitivity and scenario analyses.

These findings differ from our earlier work on this research question in the Thai HIV population where we found both pravastatin and pitavastatin would be very unlikely to be cost‐effective [17]. The main reason for this is the much lower willingness‐to‐pay threshold assumed for Thailand ($5135 per QALY gained) compared with the current analysis ($100,000 per QALY gained). We also made some methodological adjustments in this analysis, in particular, we modelled statin efficacy based on changes in LDL cholesterol as opposed to total and HDL cholesterol in the Thai study. Nevertheless, these adjustments did not substantively alter our findings (results not shown).

Heller *et al* [11] estimated that universal statin use for the primary prevention of ASCVD among men aged 45 to 74 years and women aged 55 to 74 years in the US general population would be cost‐saving. In comparison, we found expanded pravastatin use in similarly aged PLHIV less economically attractive (i.e. cost‐effective rather than cost‐saving). The main reason for this difference is Heller *et al*’s inclusion of individuals for whom statin therapy is already indicated which pushes the ICER down. Moreover, they did not account for reduced statin adherence over time. Increased survival among PLHIV is also accompanied by an increased duration of HIV management and associated costs which means, compared with the general population, life‐preserving interventions must be cheaper to achieve cost‐effectiveness.

An important consistency between our study and that of earlier US statin studies was the impact of including a quality‐of‐life decrement associated with pill burden [10, 11]. In our analysis, QALYs gained for pravastatin and pitavastatin quickly became negative compared with the no statin group when we included a small decrement in quality‐of‐life associated with remembering to take a daily statin and the inconvenience of doing so. Similarly, when a small pill burden was included in their general population model, Pandya *et al* [10] found that the optimal CVD risk score threshold for statin indication increased three‐fold (from 5% to 15% ten‐year risk) at a willingness‐to‐pay of $150,000/QALY gained. Current estimates of quality‐of‐life decrement associated with pill use vary widely [32]. Many adults in the general population may not be using other medications and could therefore experience a small quality‐of‐life decrement with having to take a daily statin pill. In contrast, PLHIV should be taking at least one ART pill per day, hence, the burden of taking an additional daily pill is likely to be small or negligible. Future research should seek to verify this assumption as it is critical to the cost‐effectiveness of expanded statin use in PLHIV.

Although pravastatin is a preferred statin in PLHIV due to its safe co‐administration with ART, it is important to note that it is not completely without ART interactions. Most notably, ritonavir‐boosted darunavir (a protease inhibitor) can moderately increase pravastatin levels [13]. Atorvastatin and rosuvastatin exhibit good efficacy in PLHIV and generic versions are priced similarly to generic pravastatin [15, 25]. Despite requiring careful dosing in combination with protease inhibitors, atorvastatin and rosuvastatin may be useful alternatives to pravastatin and our findings indicate they would be cost‐effective. We did not explicitly account for ART/statin interactions in our model because, in practice, interactions can generally be resolved by making a regimen or dose change and, although inconvenient, do not tend to cause long‐term efficacy or safety concerns.

The average improvement in cholesterol associated with statin therapy leads to about a 15% to 20% reduction in major ASCVD events [7]. Our model predicted smaller improvements because we assumed a substantial reduction in statin adherence after the first year of use, consistent with data from the general population [20, 21, 22]. It appears that PLHIV have better long‐term statin adherence than the general population [12]. However, this requires further investigation.

It remains unknown whether the anti‐inflammatory properties of statins further reduce the probability of ASCVD in PLHIV. The Randomized Trial to Prevent Vascular Events in HIV (REPRIEVE) study is currently investigating pitavastatin for the primary prevention of ASCVD in PLHIV [33]. This trial is expected to conclude in 2023 and will shed light on the overall ASCVD preventative efficacy of statins in PLHIV. However, we have shown that the cost of pitavastatin would need to drop substantially before it became cost‐effective to expand use for the primary prevention of ASCVD in PLHIV. Importantly, REPRIEVE is also investigating the impact of statin use on various non‐ASCVD outcomes, including AIDS‐defining illness, non‐AIDS‐defining cancer, renal disease and cirrhosis [33] Although there is a paucity of literature supporting the benefit of statins in preventing non‐ASCVD events, [23] such evidence could alter our main findings.

There are several limitations to this study. First, we used individual participant data from the D:A:D cohort, which is mostly comprised of PLHIV in Europe. However, the demographics and baseline characteristics of this population are similar to those of the US HIV population [34], and our modelled event rates were consistent with those reported for similarly aged US HIV cohorts [2, 3, 35]. For example in 2017, approximately 75% of the US HIV population was male and the median CD4 count was 608 cells/mm^3^, versus 71% and 650 cells/mm^3^ in our cohort. Although the racial composition of the US HIV population is different from our cohort (e.g. approximately 30% white in the United States vs. 55% in our cohort), race is not included in the D:A:D equation and hence had no bearing on our main findings. In a 2017 analysis of data from the North American AIDS Cohort Collaboration on Research and Design [35], T1MI incidence was 2.8, 5.8 and 8.8 per 1000 person years among PLHIV aged 40 to 49, 50 to 59 and ≥60 years respectively. Our base‐case model, based on a population with an initial median age of 51.1 years and with mostly low to moderate ASCVD risk, produced a T1MI incidence rate of 4.4 per 1000 person years. Moreover, the D:A:D dataset contains information on a wide range of ASCVD risk factors [36]. Most large US HIV cohorts lack information on ASCVD family history and stroke, and some primarily or exclusively enrol PLHIV of a single sex [37, 38, 39]. Second, there is evidence suggesting that the D:A:D equation underestimates CVD risk among PLHIV in the United States. However, this is based on an analysis of the HIV Outpatient Study [40] which underestimates the prevalence of ASCVD family history – a key variable in the D:A:D equation. Furthermore, we found that our main conclusions were unchanged when we used the pooled cohort equations with HIV‐associated hazard ratios to calculate T1MI and stroke risk. Third, we were not able to adjust for the use of new antiretrovirals such as integrase inhibitors as it is currently not known what impact these agents have on ASCVD risk. Finally, we had to estimate various model parameters using data from the general population due to a lack of HIV‐specific data. Most of these parameters were associated with the probability of recurrent ASCVD and the costs and quality‐of‐life estimates for ASCVD. While it is plausible that these parameters differ substantially between the general population and PLHIV, our sensitivity analyses suggested that this would have minimal impact on our main findings.

## 5 CONCLUSIONS

At a willingness‐to‐pay threshold of $100,000/QALY gained, expanding generic pravastatin use to PLHIV aged 40 to 75 years, stable on ART, and not currently using lipid‐lowering therapy was projected to be cost‐effective for the primary prevention of ASCVD. With substantial price reduction, pitavastatin may be cost‐effective compared with pravastatin. These findings bode well for the expanded use of statins among PLHIV in the United States. To gain greater confidence in these conclusions it is important to generate strong, HIV‐specific estimates on the efficacy of statins and the quality‐of‐life burden associated with taking an additional daily pill.

## COMPETING INTERESTS

DCB is supported by a National Health and Medical Research Council Early Career Fellowship (APP1140503) and has received research funding from Gilead Sciences; MGL has received unrestricted grants from Boehringer Ingelhiem, Gilead Sciences, Merck Sharp & Dohme, Bristol‐Myers Squibb, Janssen‐Cilag and ViiV HealthCare and consultancy fees from Gilead Sciences and data and safety monitoring board sitting fees from Sirtex Pty Ltd; DSK has received consulting fees from Gilead Sciences. The remaining authors have nothing to disclose.

## AUTHORS’ CONTRIBUTIONS

DCB conceived of and carried out the analysis and drafted the manuscript; ATN, AP, EB, MGL, JGK and DSK provided critical input to the design of the analysis; AP, MGL, LR, PR, AM, FB, RW, WES, AdAM, SdW, CP, CIH, JL and CS were essential in the collection of patient data; ATN oversaw the project; All authors helped draft the manuscript and have read and approved the final submission.

## ABBREVIATIONS

ART, Antiretroviral therapy; ASCVD, Atherosclerotic cardiovascular disease; D:A:D, Data‐collection on Adverse Effects of Anti‐HIV Drugs; ICER, Incremental cost‐effectiveness ratio**;** LDL, Low‐density lipoprotein; PLHIV, People living with HIV; REPRIEVE, Randomized Trial to Prevent Vascular Events in HIV; T1MI, Type 1 myocardial infarction.

## Supporting information


**Table S1**. Study population characteristics at beginning of simulation
**Table S2**. Model parameters
**Table S3**. Annual increase in systolic blood pressure (mmHg) by age and sex among individuals not using antihypertensive medication [9]
**Table S4**. Annual probability of developing diabetes by age and sex [10]
**Table S5**. Annual probability of smoking cessation by age [11]
**Table S6**. Probability of non‐CVD death by age [12,13]
**Table S7**. Annual probability of recurrent T1MI [17,24]
**Table S8**. Annual probability of recurrent ischemic stroke [25‐27]
**Table S9**. Annual probability of ischemic stroke after T1MI [25‐28]
**Table S10**. Annual probability of T1MI after ischemic stroke [24,26,29]
**Table S11**. Reduction in ASCVD probability with 1 mmol/L LDL cholesterol reduction [8]
**Table S12**. Annual cost of HIV management [30]
**Figure S1**. Core model structure
**Figure S2**. Tornado plots showing the impact of changes in model parameters on the incremental cost‐effectiveness ratio for pravastatin versus no statin
**Figure S3**. Tornado plots showing the impact of changes in model parameters on the incremental cost‐effectiveness ratio for pitavastatin versus pravastatin

## References

[1] Shah ASV , Stelzle D , Lee KK , Beck EJ , Alam S , Clifford S , et al. Global burden of atherosclerotic cardiovascular disease in people living with HIV. Circulation. 2018;138(11):1100–12. 29967196 10.1161/CIRCULATIONAHA.117.033369 PMC6221183

[2] Freiberg MS , Chang CC , Kuller LH , Skanderson M , Lowy E , Kraemer KL , et al. HIV infection and the risk of acute myocardial infarction. JAMA Intern Med. 2013;173(8):614–22. 23459863 10.1001/jamainternmed.2013.3728 PMC4766798

[3] Sico JJ , Chang CC , So‐Armah K , Justice AC , Hylek E , Skanderson M , et al. HIV status and the risk of ischemic stroke among men. Neurology. 2015;84(19):1933–40. 25862803 10.1212/WNL.0000000000001560 PMC4433456

[4] Beckman JA , Duncan MS , Alcorn CW , So‐Armah K , Butt AA , Goetz MB , et al. Association of human immunodeficiency virus infection and risk of peripheral artery disease. Circulation. 2018;138(3):255–65. 29535090 10.1161/CIRCULATIONAHA.117.032647 PMC6050082

[5] Freiberg MS , So‐Armah K . HIV and cardiovascular disease: we need a mechanism, and we need a plan. J Am Heart Assoc. 2016;4:e003411. 27013540 10.1161/JAHA.116.003411 PMC4943288

[6] Clement ME , Park LP , Navar AM , Okeke NL , Pencina MJ , Douglas PS , et al. Statin utilization and recommendations among HIV‐ and HCV‐infected veterans: a cohort study. Clin Infect Dis. 2016;63(3):407–13. 27143663 10.1093/cid/ciw289 PMC4946018

[7] Cholesterol Treatment Trialists Collaborators . The effects of lowering LDL cholesterol with statin therapy in people at low risk of vascular disease: meta‐analysis of individual data from 27 randomised trials. Lancet. 2012;380(9841):581–90. 22607822 10.1016/S0140-6736(12)60367-5 PMC3437972

[8] Eckard AR , Meissner EG , Singh I , McComsey GA . Cardiovascular disease, statins, and HIV. J Infect Dis. 2016;214 Suppl 2:S83–92. 27625435 10.1093/infdis/jiw288 PMC5021243

[9] Grundy SM , Stone NJ , Bailey AL , Beam C , Birtcher KK , Blumenthal RS , et al. 2018 AHA/ACC/AACVPR/AAPA/ABC/ACPM/ADA/AGS/APhA/ASPC/NLA/PCNA Guideline on the management of blood cholesterol: a report of the American College of Cardiology/American Heart Association Task Force on Clinical Practice Guidelines. J Am Coll Cardiol. 2019;73:e285–350. 30423393 10.1016/j.jacc.2018.11.003

[10] Pandya A , Sy S , Cho S , Weinstein MC , Gaziano TA . Cost‐effectiveness of 10‐year risk thresholds for initiation of statin therapy for primary prevention of cardiovascular disease. JAMA. 2015;314(2):142–50. 26172894 10.1001/jama.2015.6822 PMC4797634

[11] Heller DJ , Coxson PG , Penko J , Penko J , Pletcher MJ , Goldman L , et al. Evaluating the impact and cost‐effectiveness of statin use guidelines for primary prevention of coronary heart disease and stroke. Circulation. 2017;136(12):1087–98. 28687710 10.1161/CIRCULATIONAHA.117.027067 PMC5605438

[12] Youn B , Shireman TI , Lee Y , Galarraga O , Wilson IB . Trends in medication adherence in HIV patients in the US, 2001 to 2012: an observational cohort study. J Int AIDS Soc. 2019;22:e25382. 31441221 10.1002/jia2.25382 PMC6706701

[13] University of Liverpool . HIV drug interactions [cited 2020 Apr 21]. Available from: https://www.hiv‐druginteractions.org/checker

[14] Aberg JA , Sponseller CA , Ward DJ , Kryzhanovski VA , Campbell SE , Thompson MA . Pitavastatin versus pravastatin in adults with HIV‐1 infection and dyslipidaemia (INTREPID): 12 week and 52 week results of a phase 4, multicentre, randomised, double‐blind, superiority trial. Lancet HIV. 2017;4(7):e284–94. 28416195 10.1016/S2352-3018(17)30075-9

[15] Chastain DB , Stover KR , Riche DM . Evidence‐based review of statin use in patients with HIV on antiretroviral therapy. J Clin Transl Endocrinol. 2017;8:6–14. 29067253 10.1016/j.jcte.2017.01.004 PMC5651339

[16] Toribio M , Fitch KV , Sanchez L , Burdo TH , Williams KC , Sponseller CA , et al. Effects of pitavastatin and pravastatin on markers of immune activation and arterial inflammation in HIV. AIDS. 2017;31(6):797–806. 28252528 10.1097/QAD.0000000000001427 PMC5382495

[17] Boettiger DC , Newall AT , Chattranukulchai P , Chaiwarith R , Khusuwan S , Avihingsanon A , et al. Statins for atherosclerotic cardiovascular disease prevention in people living with HIV in Thailand: a cost‐effectiveness analysis. J Int AIDS Soc. 2020;23:e25494. 32562359 10.1002/jia2.25494 PMC7305414

[18] Friis‐Moller N , Ryom L , Smith C , Weber R , Reiss P , Dabis F , et al. An updated prediction model of the global risk of cardiovascular disease in HIV‐positive persons: the data‐collection on adverse effects of anti‐HIV Drugs (D:A:D) study. Eur J Prev Cardiol. 2016;23(2):214–23. 25882821 10.1177/2047487315579291

[19] Rodger AJ , Lodwick R , Schechter M , Deeks S , Amin J , Gilson R , et al. Mortality in well controlled HIV in the continuous antiretroviral therapy arms of the SMART and ESPRIT trials compared with the general population. AIDS. 2013;27(6):973–9. 23698063 10.1097/QAD.0b013e32835cae9c

[20] Benner JS , Glynn RJ , Mogun H , Neumann PJ , Weinstein MC , Avorn J . Long‐term persistence in use of statin therapy in elderly patients. JAMA. 2002;288(4):455–61. 12132975 10.1001/jama.288.4.455

[21] Colantonio LD , Rosenson RS , Deng L , et al. Adherence to statin therapy among US adults between 2007 and 2014. J Am Heart Assoc. 2019;8:e010376. 30616455 10.1161/JAHA.118.010376 PMC6405715

[22] Dorais M , Chirovsky D , Ambegaonkar B , Sazonov V , Davies G , Grant S , et al. Utilization patterns of extended‐release niacin in Canada: analysis of an administrative claims database. Can J Cardiol. 2010;26(7):e229–235. 20847969 10.1016/s0828-282x(10)70413-x PMC2950713

[23] He Y , Li X , Gasevic D , Brunt E , McLachlan F , Millenson M , et al. Statins and multiple noncardiovascular outcomes: umbrella review of meta‐analyses of observational studies and randomized controlled trials. Ann Intern Med. 2018;169(8):543–53. 30304368 10.7326/M18-0808

[24] Rosenson RS , Colantonio LD , Burkholder GA , Chen L , Muntner P . Trends in utilization of statin therapy and contraindicated statin use in HIV–infected adults treated with antiretroviral therapy from 2007 through 2015. J Am Heart Assoc. 2018;7:e010345. 30526249 10.1161/JAHA.118.010345 PMC6405602

[25] Red Book Online . Pravastatin 40mg, Pitavastatin 4mg, Atorvastatin 10mg, Rosuvastatin 10mg, and Fluvastatin 80mg wholesale acquisition costs. [cited 2019 Jan 3]. Available from: www.ibm.com/products/micromedex‐red‐book

[26] Bureau of Labor Statistics . CPI inflation calculator [cited 2019 May 05]. Available from: https://www.bls.gov/data/inflation_calculator.htm

[27] Sanders GD , Neumann PJ , Basu A , Brock DW , Feeny D , Krahn M , et al. Recommendations for conduct, methodological practices, and reporting of cost‐effectiveness analyses: second panel on cost‐effectiveness in health and medicine. JAMA. 2016;316(10):1093–103. 27623463 10.1001/jama.2016.12195

[28] Neumann PJ , Cohen JT , Weinstein MC . Updating cost‐effectiveness–the curious resilience of the $50,000‐per‐QALY threshold. N Engl J Med. 2014;371(9):796–7. 25162885 10.1056/NEJMp1405158

[29] Yadlowsky S , Hayward RA , Sussman JB , McClelland RL , Min YI , Basu S . Clinical implications of revised pooled cohort equations for estimating atherosclerotic cardiovascular disease risk. Ann Intern Med. 2018;169(1):20–9. 29868850 10.7326/M17-3011

[30] Feinstein MJ , Nance RM , Drozd DR , Ning H , Delaney JA , Heckbert SR , et al. Assessing and refining myocardial infarction risk estimation among patients with human immunodeficiency virus: a study by the centers for AIDS research network of integrated clinical systems. JAMA Cardiol. 2017;2(2):155–62. 28002550 10.1001/jamacardio.2016.4494 PMC5310962

[31] Law MG , Friis‐Moller N , El‐Sadr WM , Weber R , Reiss P , D'Arminio Monforte A , et al. The use of the Framingham equation to predict myocardial infarctions in HIV‐infected patients: comparison with observed events in the D:A: D study. HIV Med. 2006;7(4):218–30. 16630034 10.1111/j.1468-1293.2006.00362.x

[32] Fontana M , Asaria P , Moraldo M , Finegold J , Hassanally K , Manisty CH , et al. Patient‐accessible tool for shared decision making in cardiovascular primary prevention: balancing longevity benefits against medication disutility. Circulation. 2014;129(24):2539–46. 24744274 10.1161/CIRCULATIONAHA.113.007595 PMC4751622

[33] Gilbert JM , Fitch KV , Grinspoon SK . HIV‐related cardiovascular disease, statins, and the REPRIEVE trial. Top Antivir Med. 2015;23(4):146–9. 26713505 PMC6148918

[34] Centers for Disease Control and Prevention . Behavioral and clinical characteristics of persons with diagnosed HIV infection—medical monitoring project, United States, 2015‐2017 cycles [cited 2020 Apr 13]. Available from: https://www.cdc.gov/hiv/library/reports/hiv‐surveillance.html

[35] Drozd DR , Kitahata MM , Althoff KN , Zhang J , Gange SJ , Napravnik S , et al. Increased risk of myocardial infarction in HIV‐infected individuals in north America compared with the general population. J Acquir Immune Defic Syndr. 2017;75(5):568–76. 28520615 10.1097/QAI.0000000000001450 PMC5522001

[36] Friis‐Moller N , Thiebaut R , Reiss P , Weber R , D'Arminio Monforte A , De Wit S , et al. Predicting the risk of cardiovascular disease in HIV‐infected patients: the data collection on adverse effects of anti‐HIV drugs study. Eur J Cardiovasc Prev Rehabil. 2010;17(5):491–501. 20543702 10.1097/HJR.0b013e328336a150

[37] Gange SJ , Kitahata MM , Saag MS , Bangsberg DR , Bosch RJ , Brooks JT , et al. Cohort profile: the North American AIDS Cohort Collaboration on Research and Design (NA‐ACCORD). Int J Epidemiol. 2007;36(2):294–301. 17213214 10.1093/ije/dyl286 PMC2820873

[38] Justice AC , Dombrowski E , Conigliaro J , Fultz SL , Gibson D , Madenwald T , et al. Veterans Aging Cohort Study (VACS): overview and description. Med Care. 2006;44 8 Suppl 2:S13–24. 16849964 10.1097/01.mlr.0000223741.02074.66 PMC3049942

[39] Kitahata MM , Rodriguez B , Haubrich R , Boswell S , Mathews WC , Lederman MM , et al. Cohort profile: the centers for AIDS research network of integrated clinical systems. Int J Epidemiol. 2008;37(5):948–55. 18263650 10.1093/ije/dym231 PMC2597168

[40] Thompson‐Paul AM , Lichtenstein KA , Armon C , Palella FJ , Skarbinski J , Chmiel JS , et al. Cardiovascular disease risk prediction in the HIV outpatient study. Clin Infect Dis. 2016;63(11):1508–16. 27613562 10.1093/cid/ciw615 PMC5624518

