# Supplementary material for: Cost‐effectiveness of statins for primary prevention of atherosclerotic cardiovascular disease among people living with HIV in the United States
Source: J Int AIDS Soc. 2021 Mar 21;24(3):e25690. doi: 10.1002/jia2.25690 (PMC7982504; doi:10.1002/jia2.25690)
Supplement: Supplementary file 1 — Table S1. Study population characteristics at beginning of simulation Table S2. Model parameters Table S3. Annual increase in systolic blood pressure (mmHg) by age and sex among individuals not using antihypertensive medication [9] Table S4. Annual probability of developing diabetes by age and sex [10] Table S5. Annual probability of smoking cessation by age [11] Table S6. Probability of non‐CVD death by age [12,13] Table S7. Annual probability of recurrent T1MI [17,24] Table S8. Annual probability of recurrent ischemic stroke [25‐27] Table S9. Annual probability of ischemic stroke after T1MI [25‐28] Table S10. Annual probability of T1MI after ischemic stroke [24,26,29] Table S11. Reduction in ASCVD probability with 1 mmol/L LDL cholesterol reduction [8] Table S12. Annual cost of HIV management [30] Figure S1. Core model structure Figure S2. Tornado plots showing the impact of changes in model parameters on the incremental cost‐effectiveness ratio for pravastatin versus no statin Figure S3. Tornado plots showing the impact of changes in model parameters on the incremental cost‐effectiveness ratio for pitavastatin versus pravastatin [file JIA2-24-e25690-s001.docx]

**Cost-effectiveness of statins for primary prevention of atherosclerotic cardiovascular disease among people living with HIV in the United States**

- **Supplementary Material**

**Model Summary**

We used individual participant data from the Data-collection on Adverse Effects of Anti-HIV Drugs (D:A:D) study, the updated D:A:D cardiovascular disease risk equation, and published literature to estimate medical costs and quality-adjusted life-years (QALYs) among people living with HIV (PLHIV) in the US.

**Study Population**

The D:A:D study is an ongoing collaboration of 11 observational HIV cohorts in Europe, the US, Argentina and Australia.^1^ Our study population included PLHIV enrolled in D:A:D who had documentation of at least one clinic visit on or after 1 January 2013 and who, at their last documented clinic visit, were aged 40-75 years, had no history of atherosclerotic cardiovascular disease (ASCVD), were not using lipid-lowering therapy, had been using antiretroviral therapy (ART) for at least 6 months, and had a CD4 cell count >100 cells/mm^3^. Stable ART was included as a selection criterion as this should be prioritized by PLHIV over ASCVD risk management. Individuals with missing ASCVD risk factor data were excluded. Supplementary Table 1 characterizes the 4,565 D:A:D enrolees included in our study population. All cohorts in D:A:D follow local national guidelines and regulations regarding participant consent and ethical review. This analysis was approved by both the University of California, San Francisco Institutional Review Board (IRB#18-25654) and the UNSW Sydney Human Research Ethics Committee Executive (HC#180398).

**Supplementary Table 1 – Study population characteristics at beginning of simulation**

| **Characteristic** | **N=4,565** | |
| --- | --- | --- |
| Sex | Male | 3,240 (71.0) |
| Age, years | Median (IQR) | 51.1 (46.6, 56.3) |
| Race | White | 2,493 (54.6) |
|  | Non-white | 2,072 (45.4) |
| Mode of HIV exposure | Heterosexual | 2,054 (45.0) |
|  | Homosexual | 1,725 (37.8) |
|  | Intravenous drug use | 545 (11.9) |
|  | Other | 241 (5.3) |
| Hepatitis C antibody status | Positive | 965 (21.1) |
| Hepatitis B surface antigen status | Positive | 262 (5.7) |
| Family history of ASCVD | Yes | 795 (17.4) |
| Diabetic | Yes | 214 (4.7) |
| Current smoker | Yes | 1,739 (38.1) |
| Ever smoked | Yes | 3,015 (66.1) |
| Systolic blood pressure, mmHg | Median (IQR) | 125 (115, 136) |
| Using antihypertensive medication | Yes | 968 (21.2) |
| Total cholesterol, mmol/L | Median (IQR) | 5.0 (4.3, 5.7) |
| LDL cholesterol, mmol/L | Median (IQR) | 3.3 (2.7, 3.9) |
| HDL cholesterol, mmol/L | Median (IQR) | 1.3 (1.1, 1.6) |
| CD4 cell count, cells/mm^3^ | Median (IQR) | 650 (475, 852) |
| D:A:D risk score, 5-year risk of CVD | ≤1% | 439 (9.6) |
|  | >1 – 5% | 2,823 (61.8) |
|  | >5% | 1,303 (28.5) |

All values are No. (%) unless otherwise specified. IQR, interquartile range; ASCVD, atherosclerotic cardiovascular disease; LDL, low density lipoprotein; HDL, high density lipoprotein; D:A:D, Data-collection on Adverse Effects of Anti-HIV Drugs

**Model Structure and Model Parameters**

To inform the structure of our model and select appropriate parameter estimates we searched PubMed, applicable guideline documents, and abstracts presented at appropriate international conferences. Material in English published or presented after 1999 was considered potentially relevant. We conducted two distinct PubMed searches. The first term used was (“cardiovascular” or “heart” or “coronary” or “cerebrovascular” or “myocardial” or “stroke”) AND (“HIV” or “human immunodeficiency virus”) and the second term was (“statin” or “pravastatin” or “pitavastatin” or “atorvastatin” or "simvastatin" or “fluvastatin” or “lovastatin” or “rosuvastatin”) AND (“cost-effectiveness” or “cost-utility” or “cost-benefit” or “cost”). The highest level of evidence available was used to parameterize the model. Where multiple divergent estimates were available, we adopted a wide sensitivity range to include all estimates.

We developed a discrete-state microsimulation model that randomly selected (with replacement) 50,000 PLHIV from our D:A:D study population and simulated their experience over time. The model assumed the US healthcare sector perspective and applied a 20-year time horizon (with half-cycle correction) to allow sufficient event accumulation to compare treatment strategies. The model structure is depicted in Supplementary Figure 1.

Model parameters are described in detail in Supplementary Tables 2 to 12. Primary ASCVD risk was calculated using the reduced D:A:D CVD risk equation.^2^ This is the only HIV-specific risk equation recommended by the American College of Cardiology/American Heart Association for PLHIV.^3^ We used the reduced D:A:D equation (which is based on age, sex, diabetes status, family history of ASCVD, current smoking status, past smoking status, total cholesterol, high density lipoprotein cholesterol, systolic blood pressure, and CD4 count) rather than the full equation (which also includes ART) because the reduced model is recommended for PLHIV exposed to ART for more than 5 years.^2^ We modelled the effectiveness of statins through the simulated change in low density lipoprotein (LDL) cholesterol. During the first year of statin use, we assumed individuals would achieve the same reductions in LDL cholesterol observed in a recent clinical trial among PLHIV (20.5% for pravastatin; 29.7% for pitavastatin).^4^ Thereafter, statin adherence, and hence LDL lowering efficacy and associated adverse event rates, were reduced by 50%.^5-7^ The mmol/L reduction in LDL cholesterol was converted to a percentage of 1 mmol/L and the ASCVD risk reduction associated with 1 mmol/L LDL cholesterol reduction was multiplied by this percentage to ascertain each individuals ASCVD risk reduction. The reduction in ASCVD risk associated with LDL cholesterol reduction varied by individuals five-year CVD risk (based on the D:A:D equation) and the ASCVD event type being evaluated (Supplementary Table 11).^8^ We assumed PLHIV would only incur the cost of statins they were using and hence statin costs were reduced by 50% after the first year, in line with the assumed decline in adherence.

With each annual cycle we added one year of age, and assumed age- and sex-specific changes in systolic blood pressure ^9^ (Supplementary Table 3) and rates of diabetes ^10^ (Supplementary Table 4), and age-specific rates of smoking cessation (Supplementary Table 5).^11^ All other variables used to calculate ASCVD risk were kept constant over time. As the D:A:D risk score calculates five-year probability of CVD, we converted scores to rates, divided them by five and converted to one-year probabilities. Since the risk score defines CVD as a composite of coronary intervention, T1MI, stroke (ischemic or hemorrhagic) or other cardiovascular death, we apportioned the calculated risk into individual event types based on the proportions reported in Friis-Moller *et al*.^2^ The probability of non-CVD death was calculated by multiplying age and sex specific all-cause mortality estimates by age and sex specific proportions of non-CVD death in the US (Supplementary Table 6).^12,13^

Recurrent event probabilities were based on reported event rates in the years after an incident event (Supplementary Tables 7-10) and were primarily based on published estimates for the general population; we did not use the D:A:D score or an HIV-specific hazard ratio as current evidence suggests that risk factors for primary ASCVD differ substantially from those of recurrent ASCVD.^14-17^ Statins were assumed to be used as secondary ASCVD prevention in both our control and intervention arms.

Individuals accumulated costs and benefits up until the time horizon or until their death, whichever came first.

**Supplementary Figure 1 – Core model structure**

**
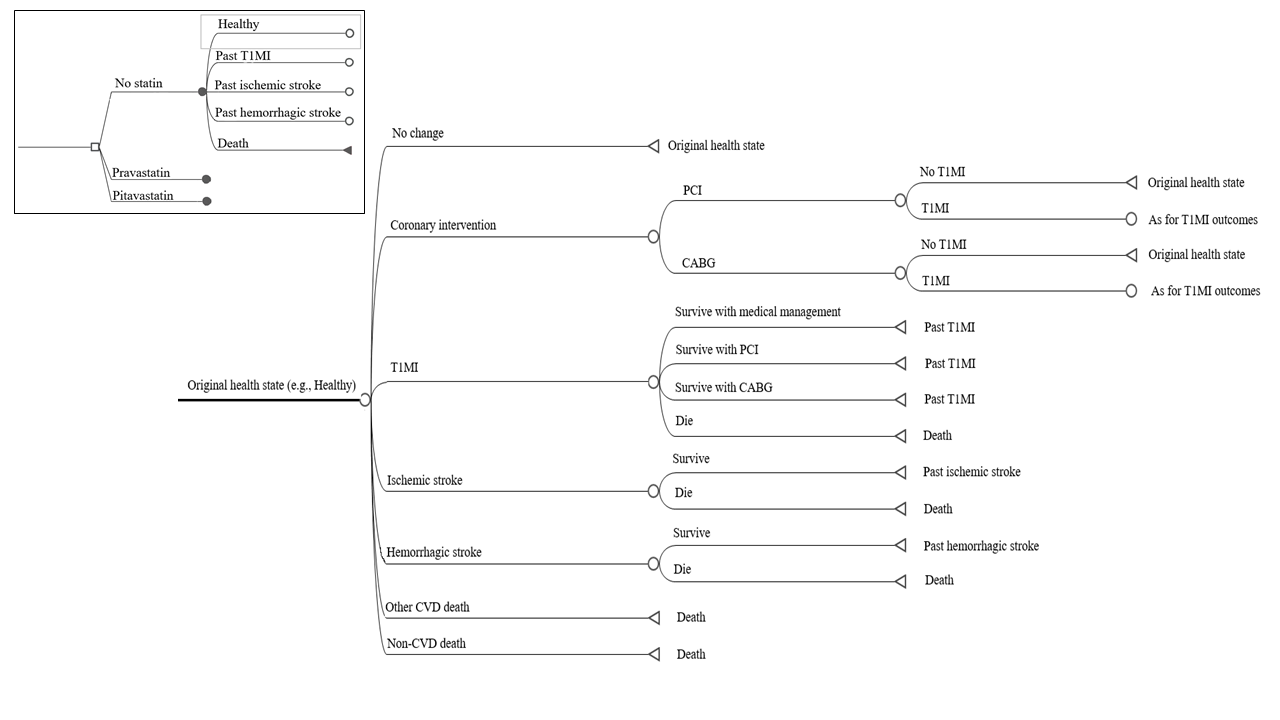
**

The box in the top left corner shows the model comprised three intervention arms branching from a decision node (unfilled square): no statin, pravastatin and pitavastatin. The Markov node (filled circle) for each intervention arm included five chronic states: healthy, past T1MI, past ischemic stroke, past hemorrhagic stroke, or death (absorbing state; filled triangle). For simplicity, the chronic states are only depicted in the sub-tree for the no statin arm. In the healthy state (highlighted by a grey rectangle in the top left box and expanded in the main tree), individuals transitioned in annual cycles to an acute state of no change, coronary intervention, T1MI, ischemic stroke, hemorrhagic stroke, other CVD death, or non-CVD death. The acute states branching from past T1MI, past ischemic stroke, and past hemorrhagic stroke are the same as those of the healthy state but without coronary intervention as an option. The unfilled circles represent chance nodes where each branch to the right of the node is assigned a pre-specified probability. The unfilled triangles represent terminal nodes and indicate where an individual ends up at the conclusion of an annual cycle and the state in which they begin the next annual cycle. Individuals accumulated costs and benefits up until their death or the time horizon, whichever came first. T1MI, type 1 myocardial infarction; CVD, cardiovascular disease; PCI, percutaneous coronary intervention; CABG, coronary artery bypass graft.

**Supplementary Table 2 – Model parameters**

| **Parameter** | **Base case**  **(range for sensitivity)** | **Source** |
| --- | --- | --- |
| ***Probabilities*** |  |  |
| *ASCVD risk factors* |  |  |
| Probability of CVD event (D:A:D equation), per year | Varies by individual^a^ | 2 |
| Probability of developing diabetes, per year | Varies by age and sex^b^ | 10 |
| Probability of smoking cessation, per year^c^ | Varies by age^b^ | 11 |
| Increase in systolic blood pressure, per year | Varies by age and sex^b^ | 9 |
| *T1MI* |  |  |
| Probability of CVD event being fatal/non-fatal T1MI | 0.488 (0.450-0.520) | 2 |
| Probability of CABG after T1MI | 0.045 (0.030-0.060) | 17 |
| Probability of PCI after T1MI | 0.632 (0.600-0.660) | 17 |
| Probability of T1MI being fatal | 0.162 (0.130-0.200) | 2 |
| *Stroke* |  |  |
| Probability of CVD event being fatal/non-fatal stroke | 0.292 (0.250-0.320) | 2 |
| Probability of stroke being ischemic | 0.800 (0.700-0.900) | 18-20 |
| Probability of ischemic stroke being fatal | 0.136 (0.090-0.200) | 2 |
| Probability of hemorrhagic stroke being fatal^c^ | 0.244 (0.174-0.322) | 21 |
| *Coronary intervention (without prior T1MI/stroke)* |  |  |
| Probability of CVD event being an intervention | 0.163 (0.140-0.190) | 2 |
| Probability of intervention being CABG | 0.218 (0.150-0.290) | 2 |
| Probability of intervention being PCI | 0.782 (0.710-0.850) | 2 |
| Probability of T1MI after CABG^c^ | 0.100 (0.050-0.300) | 22 |
| Probability of T1MI after PCI^c^ | 0.040 (0.020-0.100) | 23 |
| *Death* |  |  |
| Probability of CVD event being other cardiovascular death | 0.044 (0.030-0.060) | 2 |
| Hazard ratio of other cardiovascular death for past T1MI/stroke vs no past T1MI/stroke | 2.000 (1.000-3.000) | Assumption |
| Probability of non-CVD death, per year^c^ | Varies by age and sex^b^ | 12,13 |
| *Recurrent events* |  |  |
| Probability of recurrent T1MI, per year | Varies by age, sex and time since last T1MI^b^ | 17,24 |
| Probability of CABG after recurrent T1MI | 0.037 (0.012-0.084) | 17 |
| Probability of PCI after recurrent T1MI | 0.485 (0.399-0.572) | 17 |
| Probability that recurrent T1MI is fatal^c^ | 0.330 (0.200-0.400) | 24 |
| Probability of recurrent ischemic stroke, per year^c^ | Varies by time since last stroke^b^ | 25-27 |
| Probability that recurrent ischemic stroke is fatal^c^ | 0.270 (0.140-0.420) | 25 |
| Probability of recurrent hemorrhagic stroke in first year after initial^c^ | 0.057 (0.015-0.409) | 25 |
| Probability of recurrent hemorrhagic stroke in subsequent years, per year^c^ | Varies by individual^d^ | 25 |
| Probability that recurrent hemorrhagic stroke is fatal^c^ | 0.430 (0.070-0.930) | 25 |
| Probability of ischemic stroke after T1MI, per year^c^ | Varies by sex and time since T1MI^b^ | 25-28 |
| Probability that ischemic stroke after T1MI is fatal^c^ | 0.270 (0.140-0.420)^e^ | Assumption |
| Probability of T1MI after stroke, per year^c^ | Varies by age, sex and time since stroke^b^ | 24,26,29 |
| Probability that T1MI after stroke is fatal^c^ | 0.500 (0.250-0.750) | 29 |
| Probability of hemorrhagic stroke after T1MI, per year | Varies by individual^d^ | Assumption |
| Probability of hemorrhagic stroke after ischemic stroke, per year | Varies by individual^d^ | Assumption |
|  |  |  |
| ***Efficacy and safety of pravastatin and pitavastatin*** |  |  |
| Reduction in LDL cholesterol associated with pravastatin 40mg, % | 20.5 (5.1-35.9) | 4 |
| Reduction in LDL cholesterol associated with pitavastatin 4mg, % | 29.7 (12.3-47.1) | 4 |
| Reduction in ASCVD risk associated with 1mmol/L LDL cholesterol reduction^c^ | Varies by CVD risk and event type^b^ | 8 |
| Statin adherence after first year, % of days using statin^c^ | 50.0 (25.0-75.0) | 5-7 |
| Additional reduction in ASCVD risk associated with statin use (i.e. due to factors other than lipid change), % | 0.0 (0.0-30.0) | Assumption |
| Hazard ratio of hemorrhagic stroke for statin use vs no statin^c^ | 1.0001 (1.0000-1.0002) | 8 |
|  |  |  |
| ***Costs, 2019 $US*** |  |  |
| HIV management, per year | Varies by age and CD4^b^ | 30 |
| Non-fatal T1MI medical management^c^ | 19,586 (9,793-39,171) | 31,32 |
| PCI^c^ | 31,702 (15,851-63,402) | 31,32 |
| CABG^c^ | 106,729 (88,941-128,074) | 33-35 |
| Non-fatal T1MI management - First year post-T1MI^c^ | 13,289 (11,074-15,947) | 33-35 |
| Non-fatal T1MI management - After first year post-T1MI, per year^c^ | 2,714 (2,261-3,257) | 36 |
| Fatal T1MI^c^ | 57,693 (48,078-69,231) | 33-35 |
| Non-fatal ischemic stroke hospitalization^c^ | 21,253 (17,710-25,503) | 33-35 |
| Non-fatal ischemic stroke management - First year post-stroke^c^ | 37,387 (31,156-44,864) | 33-35 |
| Non-fatal ischemic stroke management - After first year post-stroke, per year^c^ | 5,714 (4,761-6,856) | 36 |
| Fatal ischemic stroke^c^ | 28,757 (23,964-34,508) | 33-35 |
| Non-fatal hemorrhagic stroke hospitalization^c^ | 23,616 (11,809-47,233) | 31,32 |
| Non-fatal hemorrhagic stroke management - First year post-stroke^c^ | 39,166 (19,583-78,332) | 37 |
| Non-fatal hemorrhagic stroke management - After first year post-stroke, per year^c^ | 15,997 (4,052-36,046) | 38 |
| Fatal hemorrhagic stroke^c^ | 20,405 (10,203-40,811) | 31,32 |
| Other cardiovascular death^c^ | 57,693 (48,078-69,231)^f^ | 33-35 |
| Statin-associated diabetes, average cost/individual taking statin/year^c^ | 8.26 (1.23-21.06) | 39,40 |
| Statin-associated myopathy, average cost/individual taking statin/year^c^ | 0.01 (0.00-0.02) | 41-43 |
| Pravastatin 40mg, 12-month supply | 236 (118-472)^g^ | 44 |
| Pitavastatin 4mg, 12-month supply | 2,828 (141-3,535)^h^ | 44 |
|  |  |  |
| ***Utilities*** |  |  |
| *Weights* |  |  |
| No history of CVD | 1.00 | Assumption |
| History of T1MI^c^ | 0.7780 (0.6613-0.9758) | 45-48 |
| History of ischemic stroke^c^ | 0.7680 (0.6528-0.9108) | 45-48 |
| History of hemorrhagic stroke^c,i^ | 0.6100 (0.4000-0.8000) | 49 |
| History of T1MI and ischemic stroke^c^ | 0.5975 (0.4317-0.8888) | 45-48 |
| History of T1MI and hemorrhagic stroke^c^ | 0.4746 (0.2645-0.7806) | 45-49 |
|  |  |  |
| *Acute events* |  |  |
| PCI^c^ | 0.0096 (0.0041-0.0192) | 49 |
| CABG^c^ | 0.0192 (0.0096-0.0396) | 49 |
| Acute T1MI^c^ | 0.0079 (0.0051-0.0112) | 45-47 |
| Acute ischemic stroke^c^ | 0.0113 (0.0084-0.0154) | 45-47 |
| Acute hemorrhagic stroke^c^ | 0.0113 (0.0084-0.0154)^j^ | 45-47 |
| Diabetes, average toll/individual taking statin/year^c^ | 0.00007 (0.00001-0.00019) | 40,50 |
| Myopathy, average toll/individual taking statin/year^c^ | 0.000001 (0.000000-0.000002) | 43,50 |
| Daily statin administration/pill burden^c^ | 0.00000 (0.00000-0.00384) | 38,51,52 |
| Year of age^c^ | 0.00000 (0.00000-0.00140) | 48 |
|  |  |  |
| ***Discounting and time horizon*** |  |  |
| Annual discount rate (applied to costs and benefits) | 0.03 (0.00-0.05) | 53 |
| Time horizon, years | 20 (10-30) | 53 |

^a^D:A:D equation uses age, sex, diabetes status, family history of ASCVD, current and past smoking status, total cholesterol, high density lipoprotein cholesterol, systolic blood pressure, and CD4 cell count to calculate CVD risk; ^b^See Supplementary Tables 3 to 12; ^c^Estimate based on general population data; ^d^Same as probability of incident hemorrhagic stroke calculated with D:A:D equation; ^e^Same as probability of recurrent ischemic stroke being fatal; ^f^As for fatal T1MI; ^g^Based on median wholesale acquisition cost for generic products; ^h^Based on wholesale acquisition cost; ^i^Individuals with a history of ischemic stroke and hemorrhagic stroke assumed a utility burden equivalent to hemorrhagic stroke; ^j^As for ischemic stroke; ASCVD, atherosclerotic cardiovascular disease; D:A:D, Data-collection on Adverse Effects of Anti-HIV Drugs; T1MI, type 1 myocardial infarction; CABG, coronary artery bypass graft; PCI, percutaneous coronary intervention.

**Supplementary Table 3 – Annual increase in systolic blood pressure (mmHg) by age and sex among individuals not using antihypertensive medication^9^**

| **Age, years** | **Female** | **Male** |
| --- | --- | --- |
| 40-43 | 1·3700 | 0·7400 |
| ≥44 | 1·8900 | 1·0200 |

**Supplementary Table 4 – Annual probability of developing diabetes by age and sex^10^**

| **Age, years** | **Female** | **Male** |
| --- | --- | --- |
| 40-49 | 0·0035 | 0·0088 |
| 50-59 | 0·0041 | 0·0105 |
| ≥60 | 0·0078 | 0·0198 |

**Supplementary Table 5 – Annual probability of smoking cessation by age^11^**

| **Age, years** | **Probability** |
| --- | --- |
| 40-44 | 0·0640 |
| 45-64 | 0·0490 |
| ≥65 | 0·0740 |

**Supplementary Table 6 – Probability of non-CVD death by age^12,13^**

| **Age** | **Male** | **Female** |  | **Age** | **Male** | **Female** |
| --- | --- | --- | --- | --- | --- | --- |
| 40 | 0.0020 | 0.0012 |  | 76 | 0.0263 | 0.0195 |
| 41 | 0.0021 | 0.0013 |  | 77 | 0.0291 | 0.0217 |
| 42 | 0.0022 | 0.0014 |  | 78 | 0.0321 | 0.0239 |
| 43 | 0.0022 | 0.0014 |  | 79 | 0.0349 | 0.0262 |
| 44 | 0.0024 | 0.0016 |  | 80 | 0.0385 | 0.0291 |
| 45 | 0.0025 | 0.0016 |  | 81 | 0.0424 | 0.0321 |
| 46 | 0.0027 | 0.0018 |  | 82 | 0.0466 | 0.0353 |
| 47 | 0.0029 | 0.0019 |  | 83 | 0.0516 | 0.0397 |
| 48 | 0.0031 | 0.0021 |  | 84 | 0.0566 | 0.0438 |
| 49 | 0.0034 | 0.0023 |  | 85 | 0.0624 | 0.0481 |
| 50 | 0.0036 | 0.0025 |  | 86 | 0.0684 | 0.0526 |
| 51 | 0.0039 | 0.0027 |  | 87 | 0.0755 | 0.0585 |
| 52 | 0.0043 | 0.0030 |  | 88 | 0.0839 | 0.0655 |
| 53 | 0.0047 | 0.0032 |  | 89 | 0.0918 | 0.0729 |
| 54 | 0.0051 | 0.0035 |  | 90 | 0.1003 | 0.0800 |
| 55 | 0.0056 | 0.0038 |  | 91 | 0.1092 | 0.0886 |
| 56 | 0.0060 | 0.0041 |  | 92 | 0.1202 | 0.0973 |
| 57 | 0.0065 | 0.0044 |  | 93 | 0.1287 | 0.1069 |
| 58 | 0.0070 | 0.0047 |  | 94 | 0.1389 | 0.1172 |
| 59 | 0.0074 | 0.0050 |  | 95 | 0.1504 | 0.1280 |
| 60 | 0.0080 | 0.0054 |  | 96 | 0.1610 | 0.1403 |
| 61 | 0.0087 | 0.0057 |  | 97 | 0.1720 | 0.1509 |
| 62 | 0.0093 | 0.0061 |  | 98 | 0.1822 | 0.1630 |
| 63 | 0.0098 | 0.0065 |  | 99 | 0.1945 | 0.1770 |
| 64 | 0.0104 | 0.0070 |  | 100 | 0.2048 | 0.1839 |
| 65 | 0.0111 | 0.0074 |  | 101 | 0.2220 | 0.2003 |
| 66 | 0.0118 | 0.0080 |  | 102 | 0.2406 | 0.2183 |
| 67 | 0.0126 | 0.0087 |  | 103 | 0.2608 | 0.2378 |
| 68 | 0.0136 | 0.0094 |  | 104 | 0.2827 | 0.2592 |
| 69 | 0.0146 | 0.0103 |  | 105 | 0.3065 | 0.2824 |
| 70 | 0.0156 | 0.0112 |  |  |  |  |
| 71 | 0.0171 | 0.0124 |  |  |  |  |
| 72 | 0.0183 | 0.0135 |  |  |  |  |
| 73 | 0.0201 | 0.0147 |  |  |  |  |
| 74 | 0.0218 | 0.0162 |  |  |  |  |
| 75 | 0.0239 | 0.0177 |  |  |  |  |

CVD, Cardiovascular disease

**Supplementary Table 7 – Annual probability of recurrent T1MI^17,24^**

| **Years since index T1MI** | **Male** | | | | | **Female** | | | | |
| --- | --- | --- | --- | --- | --- | --- | --- | --- | --- | --- |
|  | **30-54yo** | **55-64yo** | **65-74yo** | **75-84yo** | **>84yo** | **30-54yo** | **55-64yo** | **65-74yo** | **75-84yo** | **>84yo** |
| 1 | 0.0300 | 0.0310 | 0.0500 | 0.0900 | 0.1100 | 0.0300 | 0.0350 | 0.0500 | 0.0800 | 0.1000 |
| 4 | 0.0100 | 0.0113 | 0.0167 | 0.0300 | 0.0367 | 0.0067 | 0.0083 | 0.0200 | 0.0267 | 0.0300 |
| 7 | 0.0067 | 0.0083 | 0.0100 | 0.0100 | 0.0167 | 0.0067 | 0.0067 | 0.0067 | 0.0067 | 0.0133 |
| 30 | 0.0067 | 0.0083 | 0.0100 | 0.0100 | 0.0167 | 0.0067 | 0.0067 | 0.0067 | 0.0067 | 0.0133 |

A linear association was assumed between each data point. T1MI, type 1 myocardial infarction; yo, years old

**Supplementary Table 8 – Annual probability of recurrent ischemic stroke^25-27^**

| **Years since index ischemic stroke** | **Probability** |
| --- | --- |
| 1 | 0.0890 |
| 3 | 0.0160 |
| 5 | 0.0125 |
| 30 | 0.0125 |

A linear association was assumed between each data point

**Supplementary Table 9 – Annual probability of ischemic stroke after T1MI^25-28^**

| **Years since index T1MI** | **Male** | **Female** |
| --- | --- | --- |
| 1 | 0.0244 | 0.0427 |
| 3 | 0.0044 | 0.0077 |
| 5 | 0.0034 | 0.0060 |
| 30 | 0.0034 | 0.0060 |

A linear association was assumed between each data point. T1MI, type 1 myocardial infarction

**Supplementary Table 10 – Annual probability of T1MI after ischemic stroke^24,26,29^**

| **Years since index ischemic stroke** | **Probability** |
| --- | --- |
| 1 | 0.0220 |
| 3 | 0.0080 |
| 5 | 0.0065 |
| 30 | 0.0065 |

A linear association was assumed between each data point. T1MI, type 1 myocardial infarction

**Supplementary Table 11 – Reduction in ASCVD probability with 1mmol/L LDL cholesterol reduction^8^**

| **5-year CVD risk^a^** | **Intervention** | **T1MI** | **Ischemic stroke** | **CVD death** |
| --- | --- | --- | --- | --- |
| <5% | 0.48 | 0.43 | 0.21 | 0.38 |
| 5% to <10% | 0.37 | 0.39 | 0.21 | 0.31 |
| 10% to <20% | 0.25 | 0.23 | 0.21 | 0.21 |
| 20% to <30% | 0.21 | 0.23 | 0.21 | 0.19 |
| ≥30% | 0.24 | 0.22 | 0.21 | 0.21 |

^a^based on the D:A:D equation. ASCVD, atherosclerotic cardiovascular disease; LDL, low density lipoprotein; T1MI, type 1 myocardial infarction

**Supplementary Table 12 – Annual cost of HIV management^30^**

| **Age** | **Current CD4 cell count, cells/mm^3^** | | | |
| --- | --- | --- | --- | --- |
|  | **0-200** | **201-350** | **351-500** | **>500** |
| 35 | 38,210 | 32,475 | 29,623 | 27,922 |
| 45 | 42,076 | 35,761 | 32,620 | 30,747 |
| 55 | 45,340 | 38,535 | 35,152 | 33,133 |
| 65 | 46,764 | 39,746 | 36,256 | 34,174 |
| 75 | 48,345 | 41,089 | 37,482 | 35,329 |
| 85 | 49,681 | 42,226 | 38,518 | 36,306 |
| 95 | 50,839 | 43,210 | 39,416 | 37,152 |
| 105 | 51,860 | 44,078 | 40,208 | 37,898 |

A linear association was assumed between each data point. All costs are in 2019 dollar

**Supplementary Figure 2 – Tornado plots showing the impact of changes in model parameters on the incremental cost-effectiveness ratio for pravastatin versus no statin**

**
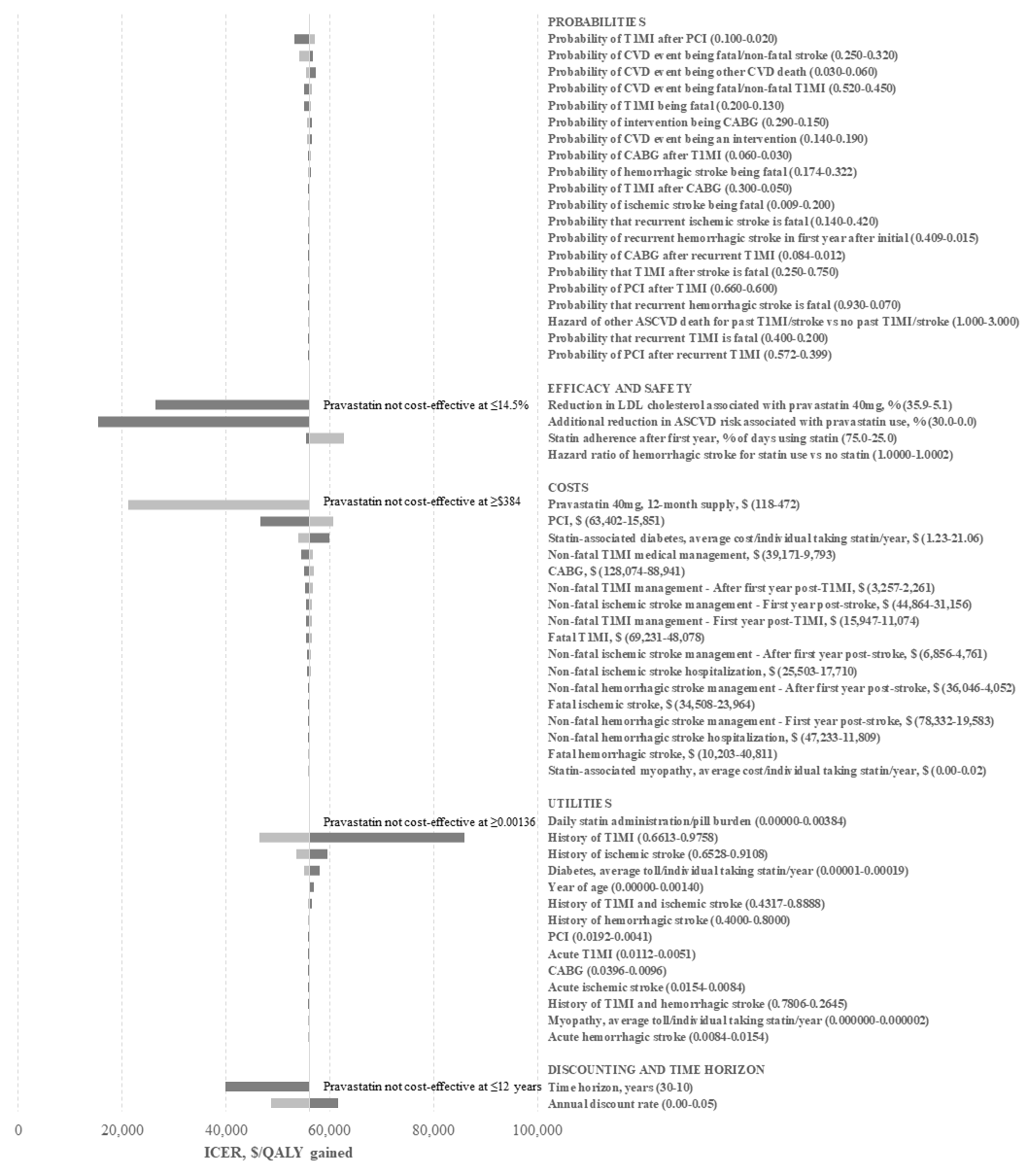
**

Shading of bars indicates directionality: lighter bars represent the smaller values in the sensitivity range and darker bars indicate the larger values. Directionality also indicated by the order of values shown in the text description. Cost-effectiveness based on a willingness-to-pay threshold of $100,000/QALY gained. T1MI, type 1 myocardial infarction; PCI, percutaneous coronary intervention; ASCVD, atherosclerotic cardiovascular disease; CABG, coronary artery bypass graft; LDL, low density lipoprotein; ICER, incremental cost-effectiveness ratio; QALY, quality-adjusted life-year

**Supplementary Figure 3 – Tornado plots showing the impact of changes in model parameters on the incremental cost-effectiveness ratio for pitavastatin versus pravastatin**


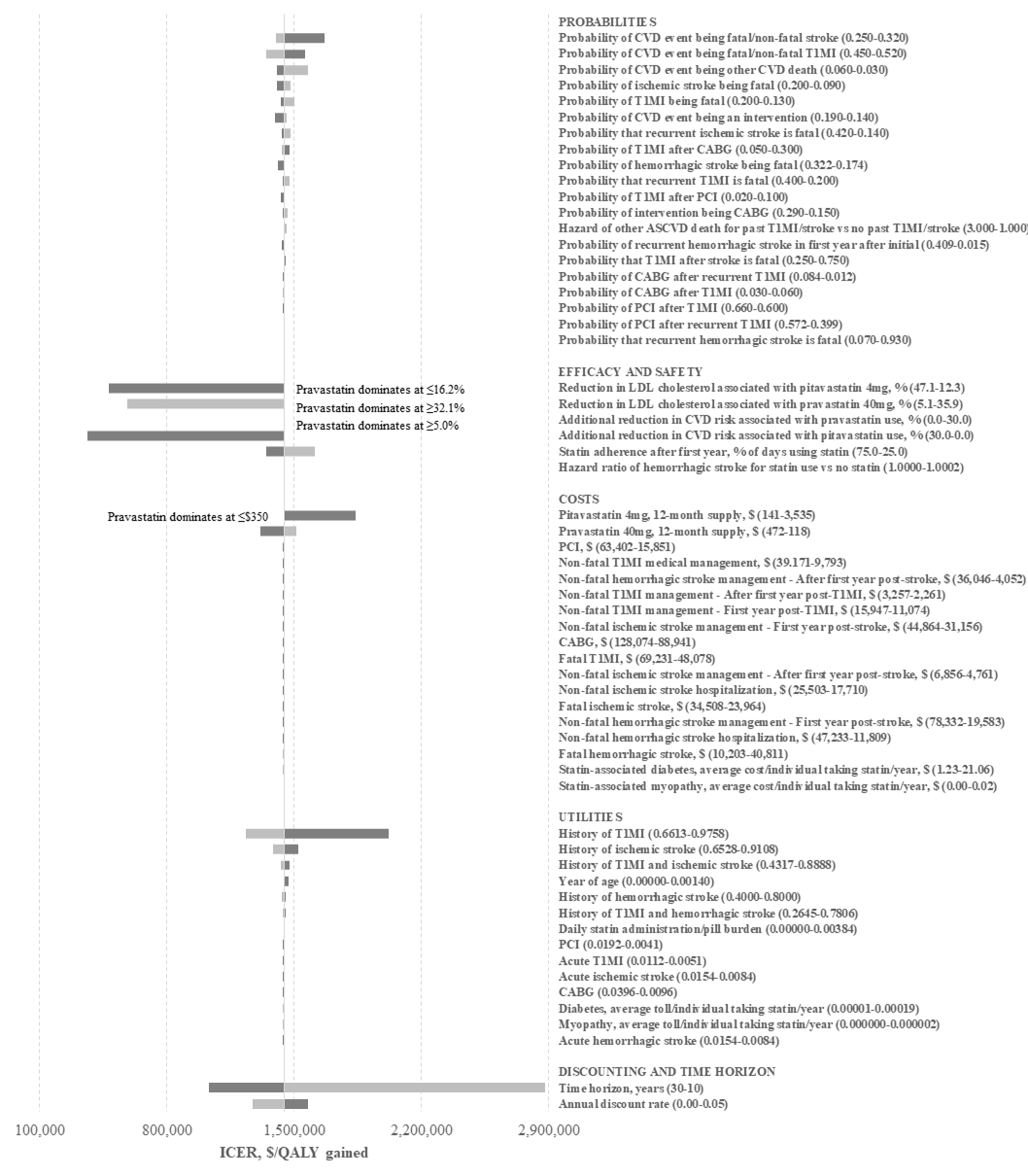


Shading of bars indicates directionality: lighter bars represent the smaller values in the sensitivity range and darker bars indicate the larger values. Directionality also indicated by the order of values shown in the text description. ASCVD, atherosclerotic cardiovascular disease; T1MI, type 1 myocardial infarction; CABG, coronary artery bypass graft; PCI, percutaneous coronary intervention; LDL, low density lipoprotein; ICER, incremental cost-effectiveness ratio; QALY, quality-adjusted life-year

**Acknowledgments D:A:D Study group**

D:A:D Participating Cohorts

Aquitaine, France; CPCRA, USA; NICE Cohort, France; ATHENA, The Netherlands; EuroSIDA, Europe; SHCS, Switzerland, AHOD, Australia; HIV-BIVUS, Sweden; St.Pierre Brussels Cohort, Belgium; BASS, Spain, The ICONA Foundation, Italy

D:A:D Steering Committee: Names marked with *, Chair with ¢

Cohort PIs: W El-Sadr* (CPCRA), G Calvo* (BASS), F Bonnet and F Dabis* (Aquitaine), O Kirk* and A Mocroft* (EuroSIDA), M Law* (AHOD), A d’Arminio Monforte* (ICONA), L Morfeldt* (HivBIVUS), C Pradier* (Nice), P Reiss* (ATHENA), R Weber* (SHCS), S De Wit* (Brussels) Cohort coordinators and data managers: A Lind-Thomsen (coordinator), R Salbøl Brandt, M Hillebreght, S Zaheri, FWNM Wit (ATHENA), A Scherrer, F Schöni-Affolter, M Rickenbach (SHCS), A Tavelli, I Fanti (ICONA), O Leleux, J Mourali, F Le Marec, E Boerg (Aquitaine), E Thulin, A Sundström (HIVBIVUS), G Bartsch, G Thompsen (CPCRA), C Necsoi, M Delforge (Brussels), E Fontas, C Caissotti, K Dollet (Nice), S Mateu, F Torres (BASS), K Petoumenos, A Blance, R Huang, R Puhr (AHOD), K Grønborg Laut, D Kristensen (EuroSIDA)

Statisticians: CA Sabin*, AN Phillips*, DA Kamara, CJ Smith, A Mocroft*

D:A:D coordinating office: CI Hatleberg, L Ryom, A Lind-Thomsen, RS Brandt, D Raben, C Matthews, A Bojesen, AL Grevsen, JD Lundgren*¢

Member of the D:A:D Oversight Committee: B Powderly*, N Shortman*, C Moecklinghoff*, G Reilly*, X Franquet*

D:A:D working group experts:

Kidney: L Ryom, A Mocroft*, O Kirk *, P Reiss*, C Smit, M Ross, CA Fux, P Morlat, E Fontas, DA Kamara, CJ Smith, JD Lundgren *¢

Mortality: CJ Smith, L Ryom, CI Hatleberg, AN Phillips*, R Weber*, P Morlat, C Pradier*, P Reiss*, FWNM Wit, N Friis-Møller, J Kowalska, JD Lundgren*¢

Cancer: CA Sabin*, L Ryom, CI Hatleberg, M Law*, A d'Arminio Monforte*, F Dabis*, F Bonnet*, P Reiss*, FWNM Wit, CJ Smith, DA Kamara, J Bohlius, M Bower, G Fätkenheuer, A Grulich, JD Lundgren*¢

External endpoint reviewers: A Sjøl (CVD), P Meidahl (oncology), JS Iversen (nephrology)

Funding: By a grant [grant number DNRF126] from the Danish National Research Foundation (CHIP & PERSIMUNE); ‘Oversight Committee for The Evaluation of Metabolic Complications of HAART’ with representatives from academia, patient community, FDA, EMA and a consortium of AbbVie, Bristol-Myers Squibb, Gilead Sciences, ViiV Healthcare, Merck and Janssen Pharmaceutic

**The current members of the 11 Cohorts are as follows:**

**ATHENA (AIDS Therapy Evaluation Project Netherlands):**

Central coordination: P. Reiss*, S. Zaheri, M Hillebregt, F.W.N.M. Wit;

CLINICAL CENTRES (¤ denotes site coordinating physician) Academic Medical Centre of the University of Amsterdam: J.M. Prins¤, T.W. Kuijpers, H.J. Scherpbier, J.T.M. van der Meer, F.W.N.M. Wit, M.H. Godfried, P. Reiss, T. van der Poll, F.J.B. Nellen, S.E. Geerlings, M. van Vugt, D. Pajkrt, J.C. Bos, W.J. Wiersinga, M. van der Valk, A. Goorhuis, J.W. Hovius, J. van Eden, A. Henderiks, A.M.H. van Hes, M. Mutschelknauss, H.E. Nobel, F.J.J. Pijnappel, S. Jurriaans, N.K.T. Back, H.L. Zaaijer, B. Berkhout, M.T.E. Cornelissen, C.J. Schinkel, X.V. Thomas. Admiraal De Ruyter Ziekenhuis, Goes: M. van den Berge, A. Stegeman, S. Baas, L. Hage de Looff, D. Versteeg. Catharina Ziekenhuis, Eindhoven: M.J.H. Pronk¤, H.S.M. Ammerlaan, E.S. de Munnik. A.R. Jansz, J. Tjhie, M.C.A. Wegdam, B. Deiman, V. Scharnhorst. Emma Kinderziekenhuis: A. van der Plas, A.M. Weijsenfeld. Erasmus MC, Rotterdam: M.E. van der Ende¤, T.E.M.S. de Vries-Sluijs, E.C.M. van Gorp, C.A.M. Schurink, J.L. Nouwen, A. Verbon, B.J.A. Rijnders, H.I. Bax, M. van der Feltz. N. Bassant, J.E.A. van Beek, M. Vriesde, L.M. van Zonneveld. A. de Oude-Lubbers, H.J. van den Berg- Cameron, F.B. Bruinsma-Broekman, J. de Groot, M. de Zeeuw- de Man, C.A.B. Boucher, M.P.G Koopmans, J.J.A van Kampen, S.D. Pas. Erasmus MC–Sophia, Rotterdam: G.J.A. Driessen, A.M.C. van Rossum, L.C. van der Knaap, E. Visser. Flevoziekenhuis, Almere: J. Branger¤, A. Rijkeboer Mes, C.J.H.M. Duijf-van de Ven. HagaZiekenhuis, Den Haag: E.F. Schippers¤, C. van Nieuwkoop. J.M. van IJperen, J. Geilings. G. van der Hut. P.F.H. Franck. HIV Focus Centrum (DC Klinieken): A. van Eeden¤. W. Brokking, M. Groot, L.J.M. Elsenburg, M. Damen, I.S. Kwa. Isala, Zwolle: P.H.P. Groeneveld¤, J.W. Bouwhuis, J.F. van den Berg, A.G.W. van Hulzen, G.L. van der Bliek, P.C.J. Bor, P. Bloembergen, M.J.H.M. Wolfhagen, G.J.H.M. Ruijs. Leids Universitair Medisch Centrum, Leiden:, F.P. Kroon¤, M.G.J. de Boer, M.P. Bauer, H. Jolink, A.M. Vollaard, W. Dorama, N. van Holten, E.C.J. Claas, E. Wessels. Maasstad Ziekenhuis, Rotterdam: J.G. den Hollander¤, K. Pogany, A. Roukens, M. Kastelijns, J.V. Smit, E. Smit, D. Struik-Kalkman, C. Tearno, M. Bezemer, T. van Niekerk, O. Pontesilli. Maastricht UMC+, Maastricht: S.H. Lowe¤, A.M.L. Oude Lashof, D. Posthouwer, R.P. Ackens, J. Schippers, R. Vergoossen, B. Weijenberg-Maes, I.H.M. van Loo, T.R.A. Havenith. MCHBronovo, Den Haag: E.M.S. Leyten¤, L.B.S. Gelinck, A. van Hartingsveld, C. Meerkerk, G.S. Wildenbeest, J.A.E.M. Mutsaers, C.L. Jansen. MC Slotervaart, Amsterdam: J.W. Mulder, S.M.E. Vrouenraets, F.N. Lauw, M.C. van Broekhuizen, H. Paap, D.J. Vlasblom, P.H.M. Smits. MC Zuiderzee, Lelystad: S. Weijer¤, R. El Moussaoui, A.S. Bosma. Medisch Centrum Leeuwarden, Leeuwarden: M.G.A.van Vonderen¤, D.P.F. van Houte, L.M. Kampschreur, K. Dijkstra, S. Faber, J Weel. Medisch Spectrum Twente, Enschede: G.J. Kootstra¤, C.E. Delsing, M. van der Burg-van de Plas, H. Heins, E. Lucas. Noorwest Ziekenhuisgroep, Alkmaar: W. Kortmann¤, G. van Twillert¤, J.W.T. Cohen Stuart, B.M.W. Diederen, D. Pronk, F.A. van Truijen-Oud, W. A. van der Reijden, R. Jansen. OLVG, Amsterdam: K. Brinkman¤, G.E.L. van den Berk, W.L. Blok, P.H.J. Frissen, K.D. Lettinga W.E.M. Schouten, J. Veenstra, C.J. Brouwer, G.F. Geerders, K. Hoeksema, M.J. Kleene, I.B. van der Meché, M. Spelbrink, H. Sulman, A.J.M. Toonen, S. Wijnands, M. Damen, D. Kwa, E. Witte. Radboudumc, Nijmegen: P.P. Koopmans, M. Keuter, A.J.A.M. van der Ven, H.J.M. ter Hofstede, A.S.M. Dofferhoff, R. van Crevel, M. Albers, M.E.W. Bosch, K.J.T. Grintjes-Huisman, B.J. Zomer, F.F. Stelma, J. Rahamat-Langendoen,D. Burger. Rijnstate, Arnhem: C. Richter¤, E.H. Gisolf, R.J. Hassing, G. ter Beest, P.H.M. van Bentum, N. Langebeek, R. Tiemessen, C.M.A. Swanink. Spaarne Gasthuis, Haarlem: S.F.L. van Lelyveld¤, R. Soetekouw, N. Hulshoff, L.M.M. van der Prijt, J. van der Swaluw, N. Bermon, W.A. van der Reijden, R. Jansen, B.L. Herpers, D.Veenendaal. Medisch Centrum Jan van Goyen, Amsterdam: D.W.M. Verhagen, M. van Wijk. St Elisabeth Ziekenhuis, Tilburg: M.E.E. van Kasteren¤, A.E. Brouwer, B.A.F.M. de Kruijf-van de Wiel, M. Kuipers, R.M.W.J. Santegoets, B. van der Ven, J.H. Marcelis, A.G.M. Buiting, P.J. Kabel. Universitair Medisch Centrum Groningen, Groningen: W.F.W. Bierman¤, H. Scholvinck, K.R. Wilting, Y. Stienstra, H. de Groot-de Jonge, P.A. van der Meulen, D.A. de Weerd, J. Ludwig-Roukema, H.G.M. Niesters, A. Riezebos-Brilman, C.C. van Leer-Buter, M. Knoester. Universitair Medisch Centrum Utrecht, Utrecht: A.I.M. Hoepelman¤, T. Mudrikova, P.M. Ellerbroek, J.J. Oosterheert, J.E. Arends, R.E. Barth, M.W.M. Wassenberg, E.M. Schadd, D.H.M. van Elst-Laurijssen, E.E.B. van Oers-Hazelzet, S. Vervoort, M. van Berkel, R. Schuurman, F. Verduyn-Lunel, A.M.J. Wensing. VUmc, Amsterdam: E.J.G. Peters¤, M.A. van Agtmael, M. Bomers, J. de Vocht, M. Heitmuller, L.M. Laan, A.M. Pettersson, C.M.J.E. Vandenbroucke-Grauls, C.W. Ang. Wilhelmina Kinderziekenhuis, UMCU, Utrecht: S.P.M. Geelen, T.F.W. Wolfs, L.J. Bont, N. Nauta. COORDINATING CENTRE P. Reiss, D.O. Bezemer, A.I. van Sighem, C. Smit, F.W.N.M. Wit., T.S. Boender, S. Zaheri, M. Hillebregt, A. de Jong, D. Bergsma, P. Hoekstra, A. de Lang, S. Grivell, A. Jansen, M.J. Rademaker, M. Raethke, R. Meijering, S. Schnörr, L. de Groot, M. van den Akker, Y. Bakker, E. Claessen, A. El Berkaoui, J. Koops, E. Kruijne, C. Lodewijk, L. Munjishvili, B. Peeck, C. Ree, R. Regtop, Y. Ruijs, T. Rutkens, L. van de Sande, M. Schoorl, A. Timmerman, E. Tuijn, L. Veenenberg, S. van der Vliet, A. Wisse, T. Woudstra, B. Tuk.

**Aquitaine Cohort (France)**

Composition du Conseil scientifique :

Coordination: F. Bonnet, F. Dabis

Scientific committee: M. Dupon, V. Gaborieau, D. Lacoste, D. Malvy, P. Mercié, P. Morlat, D. Neau, JL. Pellegrin, S. Tchamgoué, E. Lazaro, C. Cazanave, M. Vandenhende, M.O. Vareil, Y. Gérard, P. Blanco, S. Bouchet, D. Breilh, H. Fleury, I. Pellegrin, G. Chêne, R. Thiébaut, L. Wittkop, L. Wittkop, O. Leleux, S. Lawson-Ayayi, A. Gimbert, S. Desjardin, L. Lacaze-Buzy, V. Petrov-Sanchez Epidemiology and Methodology: F. Bonnet, G. Chêne, F. Dabis, R. Thiébaut, L. Wittkop

Infectious Diseases and Internal Medicine: K. André, N. Bernard, F. Bonnet, O. Caubet, L. Caunegre, C. Cazanave, I. Chossat, C. Courtault, FA. Dauchy, S. De Witte, D. Dondia, M. Dupon, P. Duffau, H. Dutronc, S. Farbos, I. Faure, H. Ferrand, V. Gaborieau, Y. Gerard, C. Greib, M. Hessamfar, Y. Imbert, D. Lacoste , P. Lataste, E. Lazaro, D. Malvy, J. Marie, M. Mechain, P. Mercié, E.Monlun, P. Morlat, D. Neau, A. Ochoa, JL. Pellegrin, T. Pistone, I. Raymond,MC. Receveur, P. Rispal, L. Sorin, S. Tchamgoué, C. Valette, MA. Vandenhende, MO. Vareil, JF. Viallard, H. Wille, G. Wirth.

Immunology: I. Pellegrin, P. Blanco

Virology: H. Fleury, Me. Lafon, P. Trimoulet, P. Bellecave, C. Tumiotto

Pharmacology: S. Bouchet, D. Breilh, F. Haramburu, G. Miremeont-Salamé

Data collection, Project Management and Statistical Analyses: MJ. Blaizeau, M. Decoin, C. Hannapier, E. Lenaud et A. Pougetoux; S. Delveaux, C. D’Ivernois, F. Diarra B. Uwamaliya-Nziyumvira, O. Leleux; F. Le Marec, Eloïse Boerg, S. Lawson-Ayayi;

IT department and eCRF development: G. Palmer, V. Conte, V. Sapparrart

**AHOD (Australian HIV Observational Database, Australia):**

Central coordination: M. Law *, K. Petoumenos, R Puhr, R Huang (Sydney, New South Wales).

Participating physicians (city, state): R. Moore, S. Edwards, J. Hoy, K. Watson, N. Roth, H Lau (Melbourne, Victoria); M Bloch, D. Baker, A. Carr, D. Cooper, (Sydney, New South Wales);M O’Sullivan (Gold Coast, Queensland), D. Nolan, G Guelfi (Perth, Western Australia).

**BASS (Spain):**

Central coordination: G. Calvo, F. Torres, S. Mateu (Barcelona);

Participating physicians (city): P. Domingo, M.A. Sambeat, J. Gatell, E. Del Cacho, J. Cadafalch, M. Fuster (Barcelona); C. Codina, G. Sirera, A. Vaqué (Badalona).

**The Brussels St Pierre Cohort (Belgium):**

Coordination: S. De Wit*, N. Clumeck, M. Delforge, C. Necsoi.

Participating physicians: N. Clumeck, S. De Wit*, AF Gennotte, M. Gerard, K. Kabeya, D.

Konopnicki, A. Libois, C. Martin, M.C. Payen, P. Semaille, Y. Van Laethem.

**CPCRA (USA):**

Central coordination: J. Neaton, G. Bartsch, W.M. El-Sadr*, E. Krum, G. Thompson, D. Wentworth;

Participating physicians (city, state): R. Luskin-Hawk (Chicago, Illinois); E. Telzak (Bronx, New York); W.M. El-Sadr (Harlem, New York); D.I. Abrams (San Francisco, California); D. Cohn (Denver, Colorado); N. Markowitz (Detroit, Michigan); R. Arduino (Houston, Texas); D. Mushatt (New Orleans, Louisiana); G. Friedland (New Haven, Connecticut); G. Perez (Newark, New Jersey); E. Tedaldi (Philadelphia, Pennsylvania); E. Fisher (Richmond, Virginia); F. Gordin (Washington, DC); L.R. Crane (Detroit, Michigan); J. Sampson (Portland, Oregon); J. Baxter (Camden, New Jersey).

**EuroSIDA (multinational)**

Steering Committee: J Gatell, B Gazzard, A Horban, I Karpov, M Losso, A d’Arminio Monforte, C Pedersen, M Ristola, A Phillips, P Reiss, J Lundgren, J Rockstroh

Chair: J Rockstroh

Study Co-leads: A Mocroft, O Kirk

Coordinating Centre Staff: O Kirk, L Peters, C Matthews, AH Fischer, A Bojesen, D Raben, D Kristensen, K Grønborg Laut, JF Larsen, D Podlekareva

Statistical Staff: A Mocroft, A Phillips, A Cozzi-Lepri, L Shepherd, A Schultze, S Amele

The multi-centre study group, EuroSIDA (national coordinators in parenthesis).

Argentina: (M Losso), M Kundro, Hospital JM Ramos Mejia, Buenos Aires.

Austria: (B Schmied), Pulmologisches Zentrum der Stadt Wien, Vienna; R Zangerle, Medical University Innsbruck, Innsbruck.

Belarus: (I Karpov), A Vassilenko, Belarus State Medical University, Minsk, VM Mitsura, Gomel State Medical University, Gomel; D Paduto, Regional AIDS Centre, Svetlogorsk.

Belgium: (N Clumeck), S De Wit, M Delforge, Saint-Pierre Hospital, Brussels; E Florence, Institute of Tropical Medicine, Antwerp; L Vandekerckhove, University Ziekenhuis Gent, Gent.

Bosnia-Herzegovina: (V Hadziosmanovic), Klinicki Centar Univerziteta Sarajevo, Sarajevo.

Croatia: (J Begovac), University Hospital of Infectious Diseases, Zagreb.

Czech Republic: (L Machala), D Jilich, Faculty Hospital Bulovka, Prague; D Sedlacek, Charles University Hospital, Plzen.

Denmark: G Kronborg,T Benfield, Hvidovre Hospital, Copenhagen; J Gerstoft, T Katzenstein, Rigshospitalet, Copenhagen; NF Møller, C Pedersen, Odense University Hospital, Odense; L Ostergaard, Skejby Hospital, Aarhus, L Wiese, Roskilde Hospital, Roskilde; L N Nielsen, Hillerod Hospital, Hillerod.

Estonia: (K Zilmer), West-Tallinn Central Hospital, Tallinn; Jelena Smidt, Nakkusosakond Siseklinik, Kohtla-Järve.

Finland: (M Ristola), I Aho, Helsinki University Central Hospital, Helsinki.

France: (J-P Viard), Hôtel-Dieu, Paris; P-M Girard, Hospital Saint-Antoine, Paris; C Pradier, E Fontas, Hôpital de l'Archet, Nice; C Duvivier, Hôpital Necker-Enfants Malades, Paris.

Germany: (J Rockstroh), Universitäts Klinik Bonn; R Schmidt, Medizinische Hochschule Hannover; O Degen, University Medical Center Hamburg-Eppendorf, Infectious Diseases Unit, Hamburg; HJ Stellbrink, IPM Study Center, Hamburg; C Stefan, JW Goethe University Hospital, Frankfurt; J Bogner, Medizinische Poliklinik, Munich; G. Fätkenheuer, Universität Köln, Cologne.

Georgia: (N Chkhartishvili) Infectious Diseases, AIDS & Clinical Immunology Research Center, Tbilisi

Greece: (P Gargalianos), G Xylomenos, K Armenis, Athens General Hospital "G Gennimatas"; H Sambatakou, Ippokration General Hospital, Athens.

Hungary: (J Szlávik), Szent Lásló Hospital, Budapest.

Iceland: (M Gottfredsson), Landspitali University Hospital, Reykjavik.

Ireland: (F Mulcahy), St. James's Hospital, Dublin.

Israel: (I Yust), D Turner, M Burke, Ichilov Hospital, Tel Aviv; E Shahar, G Hassoun, Rambam Medical Center, Haifa; H Elinav, M Haouzi, Hadassah University Hospital, Jerusalem; D Elbirt, ZM Sthoeger, AIDS Center (Neve Or), Jerusalem.

Italy: (A D’Arminio Monforte), Istituto Di Clinica Malattie Infettive e Tropicale, Milan; R Esposito, I Mazeu, C Mussini, Università Modena, Modena; F Mazzotta, A Gabbuti, Ospedale S Maria Annunziata, Firenze; V Vullo, M Lichtner, University di Roma la Sapienza, Rome; M Zaccarelli, A Antinori, R Acinapura, M Plazzi, Istituto Nazionale Malattie Infettive Lazzaro Spallanzani, Rome; A Lazzarin, A Castagna, N Gianotti, Ospedale San Raffaele, Milan; M Galli, A Ridolfo, Osp. L. Sacco, Milan.

Latvia: (B Rozentale), Infectology Centre of Latvia, Riga.

Lithuania: (V Uzdaviniene) Vilnius University Hospital Santariskiu Klinikos, Vilnius; R Matulionyte, Center of Infectious Diseases, Vilnius University Hospital Santariskiu Klinikos, Vilnius.

Luxembourg: (T Staub), R Hemmer, Centre Hospitalier, Luxembourg.

Netherlands: (P Reiss), Academisch Medisch Centrum bij de Universiteit van Amsterdam, Amsterdam.

Norway: (V Ormaasen), A Maeland, J Bruun, Ullevål Hospital, Oslo.

Poland: (B Knysz), J Gasiorowski, M Inglot, Medical University, Wroclaw; A Horban, E Bakowska, Centrum Diagnostyki i Terapii AIDS, Warsaw; R Flisiak, A Grzeszczuk, Medical University, Bialystok; M Parczewski, K Maciejewska, B Aksak-Was, Medical Univesity, Szczecin; M Beniowski, E Mularska, Osrodek Diagnostyki i Terapii AIDS, Chorzow; T Smiatacz, M Gensing, Medical University, Gdansk; E Jablonowska, E Malolepsza, K Wojcik, Wojewodzki Szpital Specjalistyczny, Lodz; I Mozer-Lisewska, Poznan University of Medical Sciences, Poznan.

Portugal: (L Caldeira), Hospital Santa Maria, Lisbon; K Mansinho, Hospital de Egas Moniz, Lisbon; F Maltez, Hospital Curry Cabral, Lisbon.

Romania: (R Radoi), C Oprea, Spitalul de Boli Infectioase si Tropicale: Dr. Victor Babes, Bucarest.

Russia: (A Panteleev), O Panteleev, St Petersburg AIDS Centre, St Peterburg; A Yakovlev, Medical Academy Botkin Hospital, St Petersburg; T Trofimora, Novgorod Centre for AIDS, Novgorod, I Khromova, Centre for HIV/AIDS & and Infectious Diseases, Kaliningrad; E Kuzovatova, Nizhny Novgorod Scientific and Research Institute of Epidemiology and Microbiology named after Academician I.N. Blokhina, Nizhny Novogrod; E Borodulina, E Vdoushkina, Samara State Medical University, Samara.

Serbia: (D Jevtovic), The Institute for Infectious and Tropical Diseases, Belgrade.

Slovenia: (J Tomazic), University Clinical Centre Ljubljana, Ljubljana.

Spain: (JM Gatell), JM Miró, Hospital Clinic Universitari de Barcelona, Barcelona; S Moreno, J. M. Rodriguez, Hospital Ramon y Cajal, Madrid; B Clotet, A Jou, R Paredes, C Tural, J Puig, I Bravo, Hospital Germans Trias i Pujol, Badalona; P Domingo, M Gutierrez, G Mateo, MA Sambeat, Hospital Sant Pau, Barcelona; JM Laporte, Hospital Universitario de Alava, Vitoria-Gasteiz.

Sweden: (K Falconer), A Thalme, A Sonnerborg, Karolinska University Hospital, Stockholm; A Blaxhult, Venhälsan-Sodersjukhuset, Stockholm; L Flamholc, Malmö University Hospital, Malmö.

Switzerland: (A Scherrer), R Weber, University Hospital Zurich; M Cavassini, University Hospital Lausanne; A Calmy, University Hospital Geneva; H Furrer, University Hospital Bern; M Battegay, University Hospital Basel; P Schmid, Cantonal Hospital St. Gallen.

Ukraine: A Kuznetsova, Kharkov State Medical University, Kharkov; G Kyselyova, Crimean Republican AIDS centre, Simferopol; M Sluzhynska, Lviv Regional HIV/AIDS Prevention and Control CTR, Lviv.

United Kingdom: (B Gazzard), St. Stephen's Clinic, Chelsea and Westminster Hospital, London; AM Johnson, E Simons, S Edwards, Mortimer Market Centre, London; A Phillips, MA Johnson, A Mocroft, Royal Free and University College Medical School, London (Royal Free Campus); C Orkin, Royal London Hospital, London; J Weber, G Scullard, Imperial College School of Medicine at St. Mary's, London; A Clarke, Royal Sussex County Hospital, Brighton; C Leen, Western General Hospital, Edinburgh.

The following centers have previously contributed data to EuroSIDA:

Infectious Diseases Hospital, Sofia, Bulgaria

Hôpital de la Croix Rousse, Lyon, France

Hôpital de la Pitié-Salpétière, Paris, France

Unité INSERM, Bordeaux, France

Hôpital Edouard Herriot, Lyon, France

Bernhard Nocht Institut für Tropenmedizin, Hamburg, Germany

1st I.K.A Hospital of Athens, Athens, Greece

Ospedale Riuniti, Divisione Malattie Infettive, Bergamo, Italy

Ospedale di Bolzano, Divisione Malattie Infettive, Bolzano, Italy

Ospedale Cotugno, III Divisione Malattie Infettive, Napoli, Italy

Dérer Hospital, Bratislava, Slovakia

Hospital Carlos III, Departamento de Enfermedades Infecciosas, Madrid, Spain

Kiev Centre for AIDS, Kiev, Ukraine

Luhansk State Medical University, Luhansk, Ukraine

Odessa Region AIDS Center, Odessa, Ukraine

**HivBivus (Sweden):**

Central coordination: L. Morfeldt, G. Thulin, A. Sundström.

Participating physicians (city): B. Åkerlund (Huddinge); K. Koppel, A. Karlsson (Stockholm); L. Flamholc, C. Håkangård (Malmö).

**The ICONA Foundation (Italy):**

BOARD OF DIRECTORS

A d’Arminio Monforte (President), A Antinori, A Castagna, F Castelli, R Cauda, G Di Perri, M Galli, R Iardino, G Ippolito, GC Marchetti, CF Perno, F von Schloesser, P Viale

SCIENTIFIC SECRETARY

A d’Arminio Monforte, A Antinori, A Castagna, F Ceccherini-Silberstein, A Cozzi-Lepri, E Girardi, S Lo Caputo, C Mussini, M Puoti

STEERING COMMITTEE

M Andreoni, A Ammassari, A Antinori, C Balotta, A Bandera, P Bonfanti, S Bonora, M Borderi, A Calcagno, L Calza, MR Capobianchi, A Castagna, F Ceccherini-Silberstein, A Cingolani, P Cinque, A Cozzi-Lepri, A d’Arminio Monforte, A De Luca, A Di Biagio, E Girardi, N Gianotti, A Gori, G Guaraldi, G Lapadula, M Lichtner, S Lo Caputo, G Madeddu, F Maggiolo, G Marchetti, S Marcotullio, L Monno, C Mussini, S Nozza, M Puoti, E Quiros Roldan, R Rossotti, S Rusconi, MM Santoro, A Saracino, M Zaccarelli.

STATISTICAL AND MONITORING TEAM

A Cozzi-Lepri, I Fanti, L Galli, P Lorenzini, A Rodano, M Shanyinde, A Tavelli

BIOLOGICAL BANK INMI

F Carletti, S Carrara, A Di Caro, S Graziano, F Petrone, G Prota, S Quartu, S Truffa

PARTICIPATING PHYSICIANS AND CENTERS

Italy A Giacometti, A Costantini, V Barocci (Ancona); G Angarano, L Monno, C Santoro (Bari); F Maggiolo, C Suardi (Bergamo); P Viale, V Donati, G Verucchi (Bologna); F Castelli, C Minardi, E Quiros Roldan (Brescia); T Quirino, C Abeli (Busto Arsizio); PE Manconi, P Piano (Cagliari); B Cacopardo, B Celesia (Catania); J Vecchiet, K Falasca (Chieti); A Pan, S Lorenzotti (Cremona); L Sighinolfi, D Segala (Ferrara); F Mazzotta, F Vichi (Firenze); G Cassola, C Viscoli, A Alessandrini, N Bobbio, G Mazzarello (Genova); C Mastroianni, V Belvisi (Latina); P Bonfanti, I Caramma (Lecco); A Chiodera, P Milini (Macerata); A d’Arminio Monforte, M Galli, A Lazzarin, G Rizzardini, M Puoti, A Castagna, G Marchetti, MC Moioli, R Piolini, AL Ridolfo, S Salpietro, C Tincati, (Milano); C Mussini, C Puzzolante (Modena); A Gori, G Lapadula (Monza); N Abrescia, A Chirianni, G Borgia, R Orlando, G Bonadies, F Di Martino, I Gentile, L Maddaloni (Napoli); AM Cattelan, S Marinello (Padova); A Cascio, C Colomba (Palermo); F Baldelli, E Schiaroli (Perugia); G Parruti, F Sozio (Pescara); G Magnani, MA Ursitti (Reggio Emilia); M Andreoni, A Antinori, R Cauda, A Cristaudo, V Vullo, R Acinapura, G Baldin, M Capozzi, S Cicalini, A Cingolani, L Fontanelli Sulekova, G Iaiani, A Latini, I Mastrorosa, MM Plazzi, S Savinelli, A Vergori (Roma); M Cecchetto, F Viviani (Rovigo); G Madeddu, P Bagella (Sassari); A De Luca, B Rossetti (Siena); A Franco, R Fontana Del Vecchio (Siracusa); D Francisci, C Di Giuli (Terni); P Caramello, G Di Perri, S Bonora, GC Orofino, M Sciandra (Torino); M Bassetti, A Londero (Udine); G Pellizzer, V Manfrin (Vicenza) G Starnini, A Ialungo(Viterbo).

**Nice HIV Cohort (France):**

Central coordination: C. Pradier*, E. Fontas, K. Dollet, C. Caissotti.

Participating physicians: P. Dellamonica, E. Bernard, J. Courjon, E. Cua, F. De Salvador-Guillouet, J.Durant, C. Etienne, S. Ferrando, V. Mondain-Miton, A. Naqvi, I. Perbost,S. Pillet , B. Prouvost-Keller, P. Pugliese, V. Rio, K. Risso, P.M. Roger.

**SHCS (Swiss HIV Cohort Study, Switzerland):**

The data are gathered by the Five Swiss University Hospitals, two Cantonal Hospitals, 15 affiliated hospitals and 36 private physicians (listed in http://www.shcs.ch/180-health-care-providers).

Members of the Swiss HIV Cohort Study:

Aubert V, Battegay M, Bernasconi E, Böni J, Braun DL, Bucher HC, Calmy A, Cavassini M, Ciuffi A, Dollenmaier G, Egger M, Elzi L, Fehr J, Fellay J, Furrer H (Chairman of the Clinical and Laboratory Committee), Fux CA, Günthard HF (President of the SHCS), Haerry D (deputy of "Positive Council"), Hasse B, Hirsch HH, Hoffmann M, Hösli I, Kahlert C, Kaiser L, Keiser O, Klimkait T, Kouyos RD, Kovari H, Ledergerber B, Martinetti G, Martinez de Tejada B, Marzolini C, Metzner KJ, Müller N, Nicca D, Pantaleo G, Paioni P, Rauch A (Chairman of the Scientific Board), Rudin C (Chairman of the Mother & Child Substudy), Scherrer AU (Head of Data Centre), Schmid P, Speck R, Stöckle M, Tarr P, Trkola A, Vernazza P, Wandeler G, Weber R*, Yerly S.

**Supplementary Material References**

1. Friis-Moller N, Sabin CA, Weber R, et al. Combination antiretroviral therapy and the risk of myocardial infarction. *N Engl J Med.* 2003;349(21):1993-2003.

2. Friis-Moller N, Ryom L, Smith C, et al. An updated prediction model of the global risk of cardiovascular disease in HIV-positive persons: The Data-collection on Adverse Effects of Anti-HIV Drugs (D:A:D) study. *Eur J Prev Cardiol.* 2016;23(2):214-223.

3. Feinstein MJ, Hsue PY, Benjamin LA, et al. Characteristics, Prevention, and Management of Cardiovascular Disease in People Living With HIV: A Scientific Statement From the American Heart Association. *Circulation.* 2019:CIR0000000000000695.

4. Aberg JA, Sponseller CA, Ward DJ, Kryzhanovski VA, Campbell SE, Thompson MA. Pitavastatin versus pravastatin in adults with HIV-1 infection and dyslipidaemia (INTREPID): 12 week and 52 week results of a phase 4, multicentre, randomised, double-blind, superiority trial. *Lancet HIV.* 2017;4(7):e284-e294.

5. Benner JS, Glynn RJ, Mogun H, Neumann PJ, Weinstein MC, Avorn J. Long-term persistence in use of statin therapy in elderly patients. *JAMA : the journal of the American Medical Association.* 2002;288(4):455-461.

6. Colantonio LD, Rosenson RS, Deng L, et al. Adherence to Statin Therapy Among US Adults Between 2007 and 2014. *J Am Heart Assoc.* 2019;8(1):e010376.

7. Dorais M, Chirovsky D, Ambegaonkar B, et al. Utilization patterns of extended-release niacin in Canada: analysis of an administrative claims database. *Can J Cardiol.* 2010;26(7):e229-235.

8. Cholesterol Treatment Trialists Collaborators. The effects of lowering LDL cholesterol with statin therapy in people at low risk of vascular disease: meta-analysis of individual data from 27 randomised trials. *Lancet.* 2012;380(9841):581-590.

9. Thiebaut R, El-Sadr WM, Friis-Moller N, et al. Predictors of hypertension and changes of blood pressure in HIV-infected patients. *Antiviral therapy.* 2005;10(7):811-823.

10. Ledergerber B, Furrer H, Rickenbach M, et al. Factors associated with the incidence of type 2 diabetes mellitus in HIV-infected participants in the Swiss HIV Cohort Study. *Clin Infect Dis.* 2007;45(1):111-119.

11. Schauer GL, Malarcher AM, Asman KJ. Trends in the Average Age of Quitting Among U.S. Adult Cigarette Smokers. *Am J Prev Med.* 2015;49(6):939-944.

12. Arias E, Xu J. United States Life Tables, 2017. *Natl Vital Stat Rep.* 2019;68(7):1-66.

13. Centers for Disease Control and Prevention. CDC WONDER. <https://wonder.cdc.gov/controller/datarequest/D76>. Accessed 10 December 2018.

14. Cao CF, Li SF, Chen H, Song JX. Predictors and in-hospital prognosis of recurrent acute myocardial infarction. *J Geriatr Cardiol.* 2016;13(10):836-839.

15. Gao M, Zheng Y, Zhang W, Cheng Y, Wang L, Qin L. Non-high-density lipoprotein cholesterol predicts nonfatal recurrent myocardial infarction in patients with ST segment elevation myocardial infarction. *Lipids Health Dis.* 2017;16(1):20.

16. Nakashima H, Mashimo Y, Kurobe M, Muto S, Furudono S, Maemura K. Impact of Morning Onset on the Incidence of Recurrent Acute Coronary Syndrome and Progression of Coronary Atherosclerosis in Acute Myocardial Infarction. *Circ J.* 2017;81(3):361-367.

17. Sabin CA, Ryom L, d'Arminio Monforte A, et al. Abacavir use and risk of recurrent myocardial infarction. *Aids.* 2018;32(1):79-88.

18. Benjamin LA, Bryer A, Lucas S, et al. Arterial ischemic stroke in HIV: Defining and classifying etiology for research studies. *Neurol Neuroimmunol Neuroinflamm.* 2016;3(4):e254.

19. Ortiz G, Koch S, Romano JG, Forteza AM, Rabinstein AA. Mechanisms of ischemic stroke in HIV-infected patients. *Neurology.* 2007;68(16):1257-1261.

20. Tipping B, de Villiers L, Wainwright H, Candy S, Bryer A. Stroke in patients with human immunodeficiency virus infection. *Journal of neurology, neurosurgery, and psychiatry.* 2007;78(12):1320-1324.

21. Koton S, Schneider AL, Rosamond WD, et al. Stroke incidence and mortality trends in US communities, 1987 to 2011. *JAMA : the journal of the American Medical Association.* 2014;312(3):259-268.

22. Thielmann M, Sharma V, Al-Attar N, et al. ESC Joint Working Groups on Cardiovascular Surgery and the Cellular Biology of the Heart Position Paper: Perioperative myocardial injury and infarction in patients undergoing coronary artery bypass graft surgery. *European heart journal.* 2017;38(31):2392-2407.

23. Fokkema ML, James SK, Albertsson P, et al. Outcome after percutaneous coronary intervention for different indications: long-term results from the Swedish Coronary Angiography and Angioplasty Registry (SCAAR). *EuroIntervention.* 2016;12(3):303-311.

24. Smolina K, Wright FL, Rayner M, Goldacre MJ. Long-term survival and recurrence after acute myocardial infarction in England, 2004 to 2010. *Circ Cardiovasc Qual Outcomes.* 2012;5(4):532-540.

25. Callaly E, Ni Chroinin D, Hannon N, et al. Rates, Predictors, and Outcomes of Early and Late Recurrence After Stroke: The North Dublin Population Stroke Study. *Stroke; a journal of cerebral circulation.* 2016;47(1):244-246.

26. Edwards JD, Kapral MK, Fang J, Swartz RH. Long-term morbidity and mortality in patients without early complications after stroke or transient ischemic attack. *CMAJ.* 2017;189(29):E954-E961.

27. Lovett JK, Coull AJ, Rothwell PM. Early risk of recurrence by subtype of ischemic stroke in population-based incidence studies. *Neurology.* 2004;62(4):569-573.

28. Benjamin EJ, Blaha MJ, Chiuve SE, et al. Heart Disease and Stroke Statistics-2017 Update: A Report From the American Heart Association. *Circulation.* 2017;135(10):e146-e603.

29. Touze E, Varenne O, Chatellier G, Peyrard S, Rothwell PM, Mas JL. Risk of myocardial infarction and vascular death after transient ischemic attack and ischemic stroke: a systematic review and meta-analysis. *Stroke; a journal of cerebral circulation.* 2005;36(12):2748-2755.

30. Schackman BR, Fleishman JA, Su AE, et al. The lifetime medical cost savings from preventing HIV in the United States. *Med Care.* 2015;53(4):293-301.

31. Healthcare Cost and Utilization Project. Overview of the Nationwide Inpatient Sample. <www.hcup-us.ahrq.gov/nisoverview.jsp>. Accessed 1 Jan 2011.

32. U.S. Department of Commerce Bureau of Economic Analysis. National Economic Accounts: Gross Domestic Product. <www.bea.gov/national/index.htm>. Accessed 10 Dec 2013.

33. Office of Statewide Health Planning and Development. Hospital Financial Data, 1999-2000. <http://www.oshpd.ca.gov/HID/Hospital-Financial.asp#Profile>. Accessed 1 Jan 2016.

34. Office of Statewide Health Planning and Development. *California Public Patient Discharge Data.* Sacramento, CA:2008.

35. US Census Bureau. *Statistical Abstract of the United States: Average Cost to Community Hospitals Per Patient, by State (Table 204).* Washington, DC: Government Printing Office; 1998.

36. Agency for Healthcare Research and Quality. Medical Expenditure Panel Survey public use files 1998-2008. <http://meps.ahrq.gov/mepsweb/>. Accessed 1 Jan 2015.

37. Wang G, Zhang Z, Ayala C, Dunet DO, Fang J, George MG. Costs of hospitalization for stroke patients aged 18-64 years in the United States. *Journal of stroke and cerebrovascular diseases : the official journal of National Stroke Association.* 2014;23(5):861-868.

38. Heller DJ, Coxson PG, Penko J, et al. Evaluating the Impact and Cost-Effectiveness of Statin Use Guidelines for Primary Prevention of Coronary Heart Disease and Stroke. *Circulation.* 2017;136(12):1087-1098.

39. American Diabetes Association. Economic Costs of Diabetes in the U.S. in 2012. 2013;36:1033-1046.

40. Sattar N, Preiss D, Murray HM, et al. Statins and risk of incident diabetes: a collaborative meta-analysis of randomised statin trials. *Lancet.* 2010;375(9716):735-742.

41. Center for Medicare and Medicaid. Services Fee Schedule. <https://www.cms.gov/Medicare/Medicare-Fee-for-Service-Payment/FeeSchedule-GenInfo/index.html>. Accessed 26 Jun 2017.

42. Center for Medicare and Medicaid. Physician Fee Schedule. <https://www.cms.gov/apps/physician-fee-schedule/overview.aspx>. Accessed 26 Jun 2017.

43. Stone NJ, Robinson JG, Lichtenstein AH, et al. 2013 ACC/AHA guideline on the treatment of blood cholesterol to reduce atherosclerotic cardiovascular risk in adults: a report of the American College of Cardiology/American Heart Association Task Force on Practice Guidelines. *J Am Coll Cardiol.* 2014;63(25 Pt B):2889-2934.

44. Red Book Online. Pravastatin 40mg, Pitavastatin 4mg, Atorvastatin 10mg, Rosuvastatin 10mg, and Fluvastatin 80mg wholesale acquisition costs. Accessed 3 January 2019.

45. Moran AE, Forouzanfar MH, Roth GA, et al. The global burden of ischemic heart disease in 1990 and 2010: the Global Burden of Disease 2010 study. *Circulation.* 2014;129(14):1493-1501.

46. Moran AE, Forouzanfar MH, Roth GA, et al. Temporal trends in ischemic heart disease mortality in 21 world regions, 1980 to 2010: the Global Burden of Disease 2010 study. *Circulation.* 2014;129(14):1483-1492.

47. Murray CJ, Vos T, Lozano R, et al. Disability-adjusted life years (DALYs) for 291 diseases and injuries in 21 regions, 1990-2010: a systematic analysis for the Global Burden of Disease Study 2010. *Lancet.* 2012;380(9859):2197-2223.

48. Sullivan PW, Ghushchyan V. Preference-Based EQ-5D index scores for chronic conditions in the United States. *Med Decis Making.* 2006;26(4):410-420.

49. Kazi DS, Garber AM, Shah RU, et al. Cost-effectiveness of genotype-guided and dual antiplatelet therapies in acute coronary syndrome. *Ann Intern Med.* 2014;160(4):221-232.

50. Global Burden of Disease Collaborators. Global, regional, and national incidence, prevalence, and years lived with disability for 354 diseases and injuries for 195 countries and territories, 1990-2017: a systematic analysis for the Global Burden of Disease Study 2017. *Lancet.* 2018;392(10159):1789-1858.

51. Fontana M, Asaria P, Moraldo M, et al. Patient-accessible tool for shared decision making in cardiovascular primary prevention: balancing longevity benefits against medication disutility. *Circulation.* 2014;129(24):2539-2546.

52. Taylor F, Huffman MD, Macedo AF, et al. Statins for the primary prevention of cardiovascular disease. *The Cochrane database of systematic reviews.* 2013(1):CD004816.

53. Sanders GD, Neumann PJ, Basu A, et al. Recommendations for Conduct, Methodological Practices, and Reporting of Cost-effectiveness Analyses: Second Panel on Cost-Effectiveness in Health and Medicine. *JAMA : the journal of the American Medical Association.* 2016;316(10):1093-1103.
